# Stereoview Images of Hydrogen-Bonded Quinoxalines with a Helical Axis; Pyrimidines and a Pyridazine That Form Extended Tapes

**DOI:** 10.3390/ijms252212329

**Published:** 2024-11-17

**Authors:** Michael John Plater, William T. A. Harrison

**Affiliations:** Department of Chemistry, University of Aberdeen, Meston Walk, Aberdeen AB24 3UE, UK

**Keywords:** helical, pincer, hydrogen bond, supramolecular, crystallography

## Abstract

Different supramolecular motifs are formed by the crystallisation of amino-substituted derivatives of quinoxaline, pyrimidine and pyridazine. These were made from the corresponding mono- or dichlorinated heterocycles by a nucleophilic displacement reaction. The pyridine-type nitrogen atoms activate the chlorine atoms because they can stabilise a negative charge, which forms when the amine attacks the ring. One amino group can be attached under mild conditions in hot ethanol or acetonitrile, but the first then deactivates the ring so the second requires more forceful conditions using a pressure vessel at 150 °C. Butylamine is frequently used because it reduces the polarity of the product, making it easier to purify and isolate. The extended structure of the quinoxaline derivatives **16**–**18** show a common ‘pincer’ hydrogen-bond *motif*, with a quinoxaline nitrogen atom accepting two N–H···N hydrogen bonds, giving a spiral or helical axis. The chain symmetries are 4_1_, 2_1_ and 3_1_, respectively, depending on the substituents. A stereoview of each is shown. The pyrimidine derivatives **19**, **12**, **20**, **14** and **21** form hydrogen-bonded tapes and compound **20** forms inversion dimers.

## 1. Introduction

**Figure 1:**
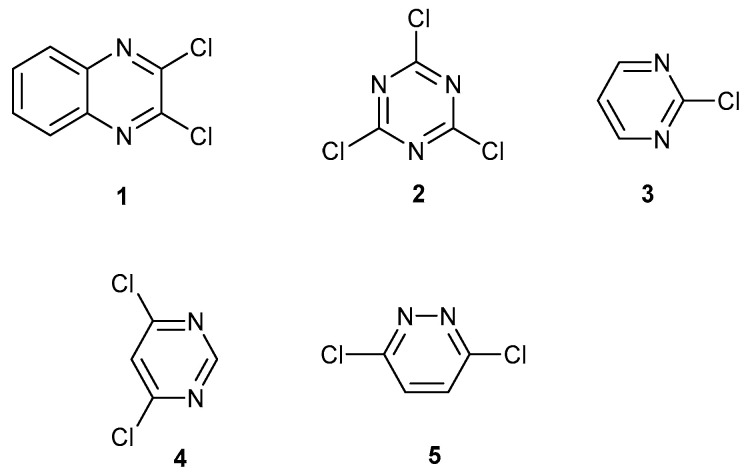
Quinoxaline, triazine, pyrimidine and pyridazine with activated chlorine atoms.

Sequential nucleophilic aromatic substitution reactions of activated halogens [1,2] and nitro groups [3] are useful for building new functionalized ring systems, particularly phenoxazines, phenothiazines, phenoxothiazines and diastereoisomers from them [3]. Heterocyclic ring systems are important as building blocks for new pharmaceuticals and general methods for their synthesis are useful [4,5,6]. They also provide model analogues for crystalline forms of pharmaceutical ingredients [7]. A wide spectrum of building blocks with activated chlorine atoms are known, which can be made or purchased. These include 2,3-dichloroquinoxaline **1** [8,9,10,11], trichlorotriazine **2** [12], 2-chloropyrimidine **3** [13,14,15], 4,6-dichloropyrimidine **4** [16,17] and 3,6-dichloropyridazine **5** [18,19,20] (Figure 1).

**Figure 2:**
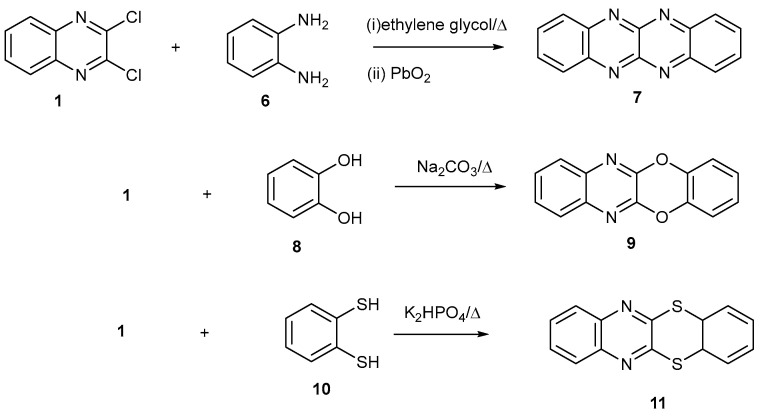
Some tetracyclic heterocycles made from 2,3-dichloroquinoxaline **1**.

Tetraazaacene **7** was made by reacting 2,3-dichloroquinoxaline **1** with *ortho*-phenylenediamine **6** followed by oxidation with PbO_2_ [21]. Compound **9** was made by reacting 2,3-dichloroquinoxaline **1** with catechol **8** [22,23] and compound **11** was prepared by reaction with benzene-1,2-dithiol [24] (Figure 2).

**Figure 3:**
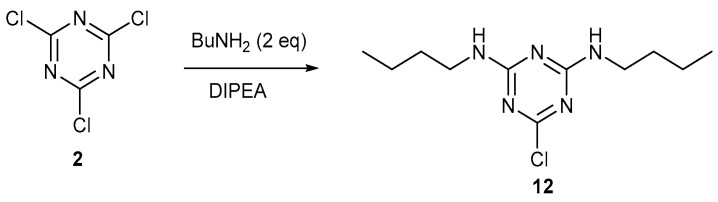
Synthesis of 2,4-bis(butylamino)-6-chloro-1,3,5-triazine **12**.

Two of the chlorine atoms of 1,3,5-trichlorotriazine **2** can be selectively displaced using butylamine (Figure 3) to give the disubstituted compound **12** [25].

**Figure 4:**
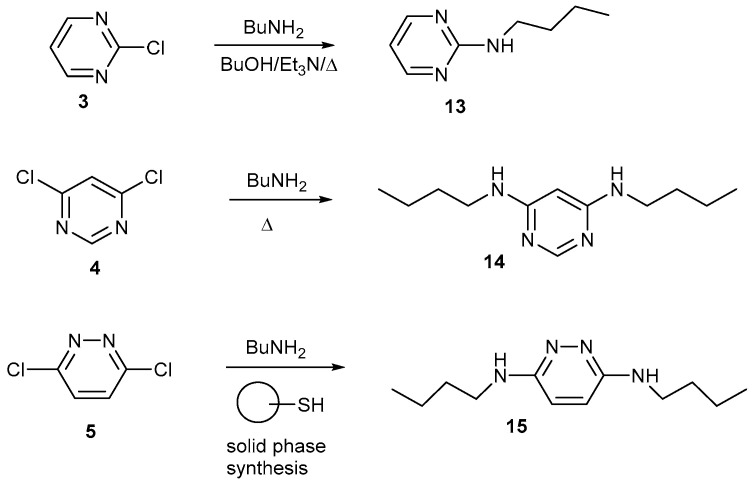
Reactions of compounds **3**–**5** with butylamine.

Figure 4 shows some further reactions of 2-chloropyrimidine **3**, 4,6-dichloropyrimidine **4** and 3,6-dichloropyrimidine **5** with butylamine. The products **13** [26], **14** [27,28] and **15** [29] are relatively easy to form. The butylamino group has been of interest to us for some time because the alkyl chain is short enough to usually be ordered in the solid state, whereas longer chains frequently lead to disorder in crystal structures [30]. The butyl chain is also long enough to reduce the polarity of the product making it easier to isolate. We have found that polar compounds are harder to isolate and purify: in some cases, we prepared compounds using a butylamino side chain, but with propyl, ethyl or methylamino side groups, no products could be isolated.

## 2. Results and Discussion

**Figure 5:**
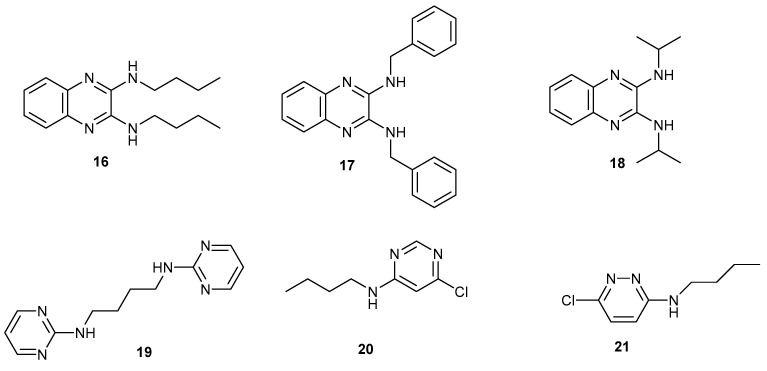
Amino derivatives of quinoxaline, pyrimidine and pyridazine.

**Figure 6:**
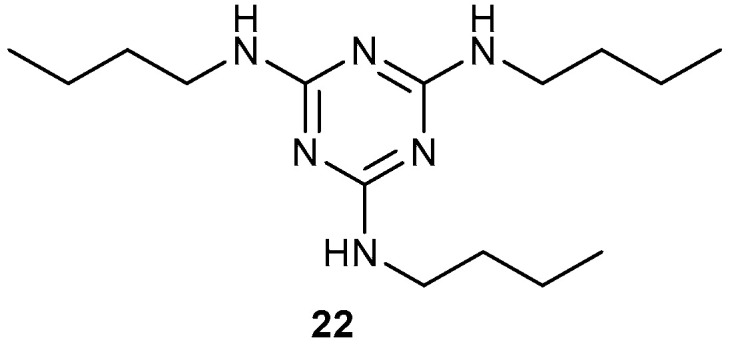
Drawing of tributylaminotriazine [an oil from DCM].

Compounds **16** [1,2]–**21** were made by similar methods, which are detailed in the experimental section (Figure 5). The syntheses of compounds **12** (Figure 3) and **14** (Figure 4) have already been reported, although the crystal structures described below are new. The diamino-substituted products **14**, **17** and **18** were prepared in pressure vessels because the higher temperature of 150 °C helps to drive the reaction to completion. An attempt was made to crystallise tributylaminotriazine **22** [31], but in our hands, only an oil was obtained from dichloromethane solution (Figure 6). Its structure was verified from its NMR data.

It was of interest to crystallise these compounds and to carry out X-ray single crystal structure determinations. Eight compounds in total were analysed. We anticipated the formation of different types of supramolecular hydrogen-bonded networks depending on the relative locations of the NH groups with respect to the ring N atoms and the possibility of weak C–H···N hydrogen bonds [32,33] similar to the C–H···O bonds that occur in biomolecules [34,35,36,37,38]. In addition, understanding how molecules pack in the solid state guided by hydrogen bonding is of interest in crystal engineering. Compounds **16**–**19** have higher molecular symmetries, which may correlate with the packing motifs of their extended architectures.

**Figure 7:**
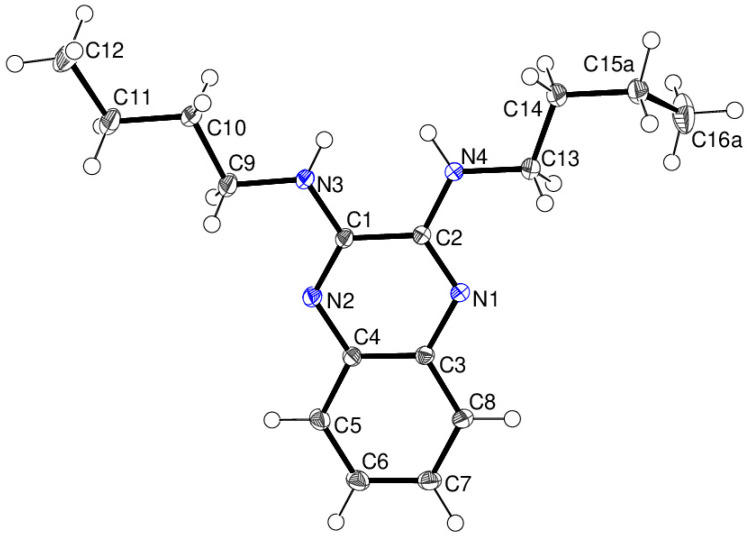
The molecular structure of compound **16** showing 50% displacement ellipsoids. Only the major orientation of the disordered C13–C16 butyl chain is shown.

Compound **16** crystallises in the tetragonal space group *I*4_1_/*a* with one molecule in the asymmetric unit (Figure 7) and the C9–C12 butyl chain adopts an extended conformation. The atoms of the terminal ethyl group of the C13–C16 chain are positionally disordered over two sets of sites in a 0.706 (5):0.294 (5) ratio. It shows a *gauche* (or *syn*) conformation with a C13—C14—C15a—C16a torsion angle of 62.8 (2)°. The quinoline ring system is slightly puckered with a dihedral angle of 3.94 (5)° between the C1–C4/N1/N2 and C3–C8 rings.

**Figure 8:**
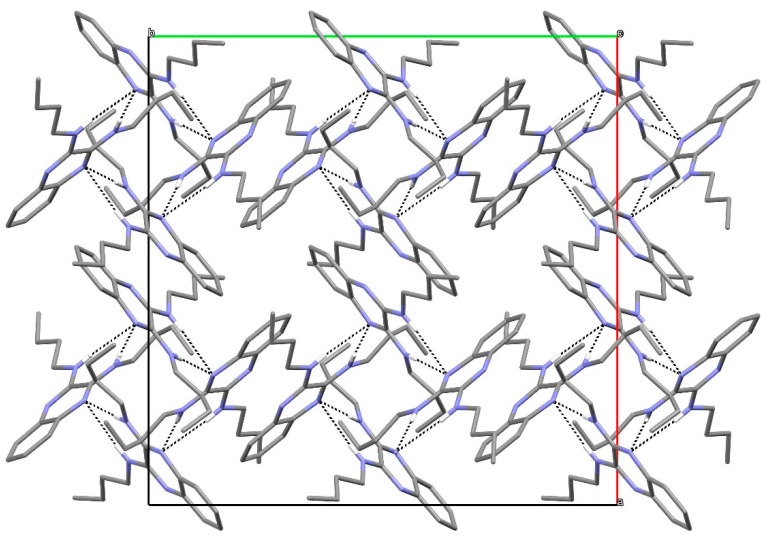
Unit-cell packing for compound **16** viewed down [001] showing the 4_1_-symmetry helical columns of molecules propagating towards the viewer. Carbon-bound hydrogen atoms are omitted for clarity and columnar hydrogen bonds are shown as black dashed lines. The red and green components of the unit-cell outline indicate the crystallographic [100] and [010] directions, respectively.

**Figure 9:**
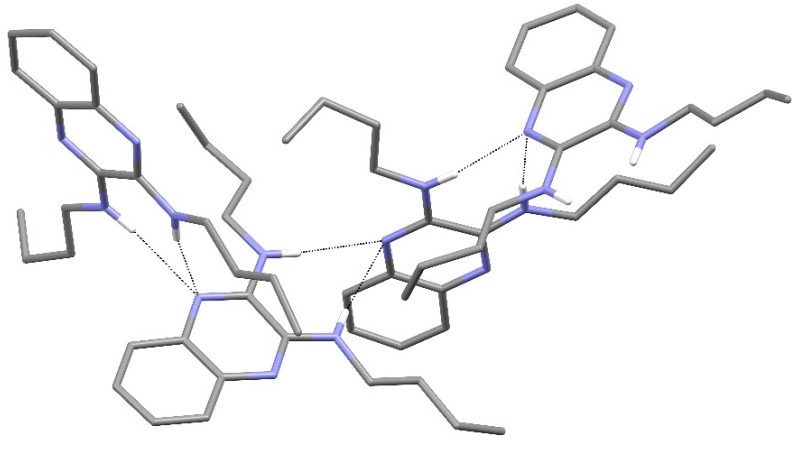
Detail of the packing of **16** showing a side-on view of the spiral hydrogen-bonded chain.

In the extended structure of compound **16**, helical hydrogen-bonded chains occur, with the quinoline N1 atom acting as a double acceptor for both N–H groups of an adjacent molecule in what we call a ‘pincer’ *motif*. The crystallographic symmetry of the column is a 4_1_ screw axis (Figure 8 and Figure 9). Due to inversion symmetry, the columns propagate both ‘up’ and ‘down’ the *c* axis direction.

**Figure 10:**
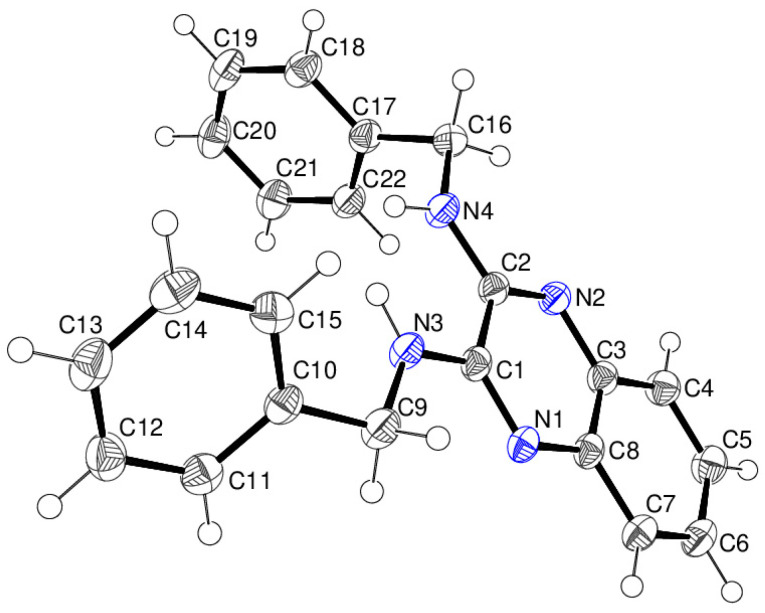
The molecular structure of the C1 molecule of compound **17** showing 50% displacement ellipsoids.

Compound **17** crystallises with two molecules (containing C1 and C23) in the asymmetric unit of the monoclinic space group *P*2_1_/*c*. In the C1 molecule (Figure 10), the C1—N3—C9—C10 moiety is *anti* (*trans*) [torsion angle = −170.65 (13)°], whereas the C2—N4—C16—C17 grouping has an anticlinal conformation [114.79 (15)°]. The dihedral angles between the C1–C8/N1/N2 fused ring system and the pendant C10–C15 and C17–C22 rings are 53.21 (4) and 84.93 (4)°, respectively. Equivalent data for the C23 molecule are C23—N7—C31—C32 = 159.77 (13)° and C24—N8—C38—C39 = 124.13 (14)°. The dihedral angles for the pendant phenyl rings in the second molecule are almost the same [78.82 (4) and 78.44 (4)°], indicating a definite conformational difference to the first molecule.

**Figure 11:**
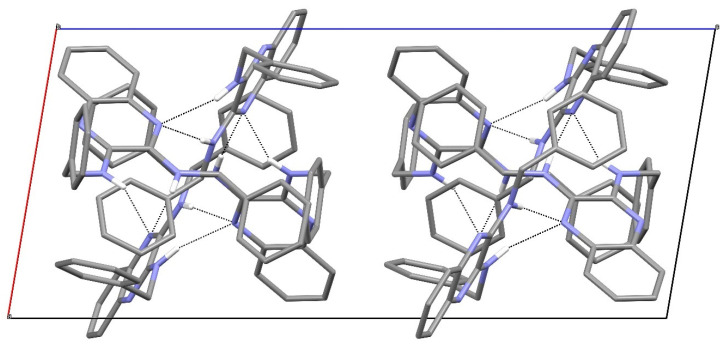
Unit-cell packing for compound **17** viewed down [010] with the 2_1_-symmetry chains of hydrogen-bonded molecules propagating towards the viewer. Carbon-bound hydrogen atoms are omitted for clarity and hydrogen bonds are shown as black dashed lines. The red and blue components of the unit-cell outline indicate the crystallographic [100] and [001] directions, respectively.

The extended structure of compound **17** features [010] hydrogen-bonded chains of alternating C1 and C23 molecules with the same pincer (double acceptor) *motif* as in compound **16**: the crystallographic 2_1_ screw axis generates the chain topology (Figure 11).

**Figure 12:**
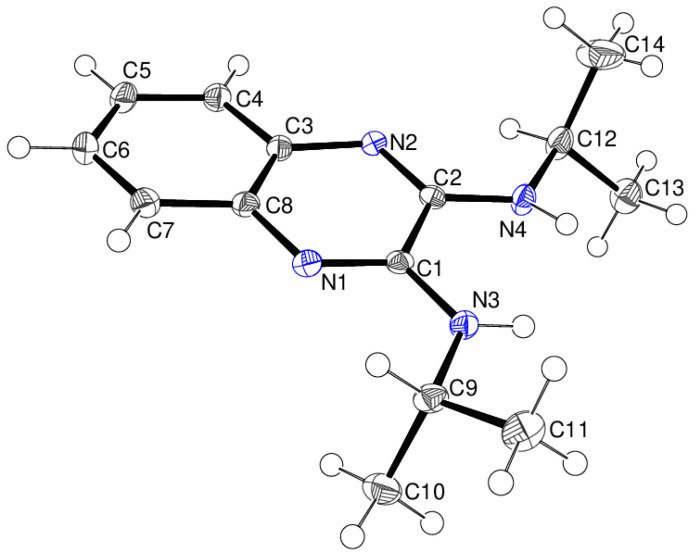
The molecular structure of the C1 molecule of compound **18** showing 50% displacement ellipsoids.

Compound **18** crystallises in the uncommon trigonal space group *P*3_1_ with two molecules (containing C1 and C15) in the asymmetric unit. In the C1 molecule (Figure 12), the key torsion angles are: C1—N3—C9—C10 = 81.0 (2)°; C1—N3—C9—C11 = −154.66 (17)°; C2—N4—C12—C13 = −143.62 (18)°; and C2—N4—C12—C14 = 92.4 (2)°.

**Figure 13:**
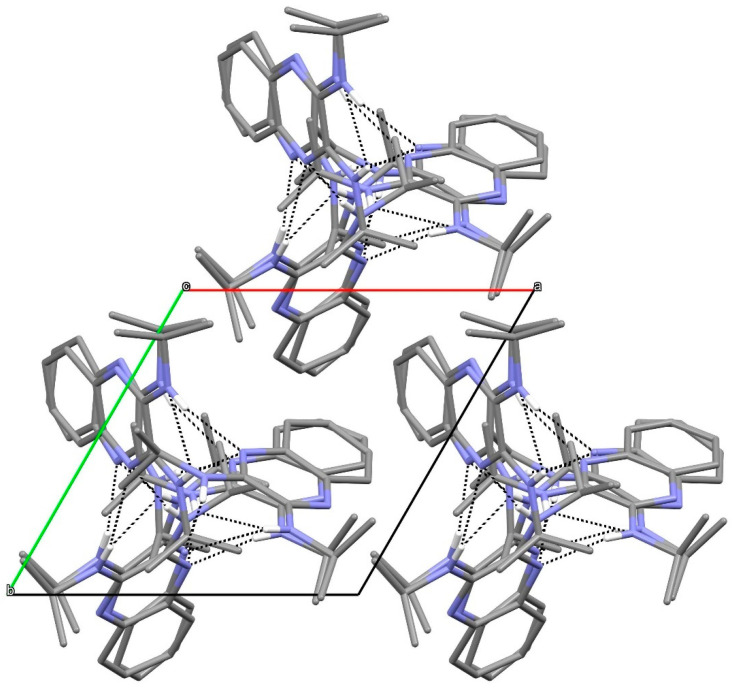
The unit-cell packing for compound **18** viewed down [001] showing the 3_1_-symmetry helical columns of molecules propagating towards the viewer. Carbon-bound hydrogen atoms are omitted for clarity and hydrogen bonds are shown as black dashed lines. The red and green components of the unit-cell outline indicate the crystallographic [100] and [010] directions, respectively.

The packing in compound **18** features [001] helical chains of alternating C1 and C15 molecules with 3_1_ symmetry (Figure 13). Inter-chain packing appears to be via van der Waals’ forces.

**Figure 14:**
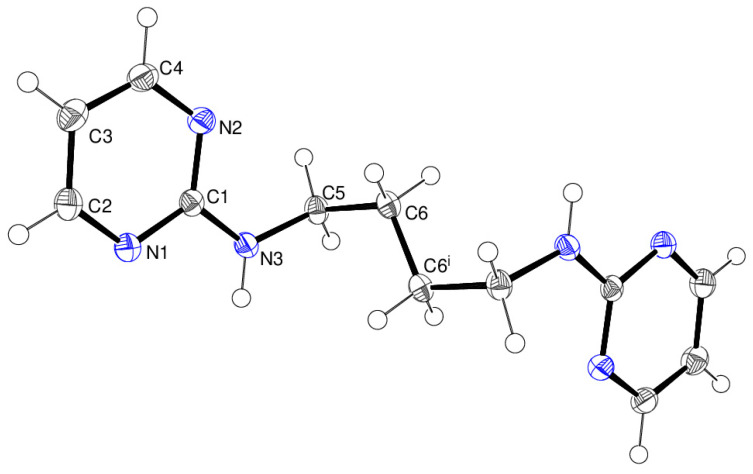
The molecular structure of compound **19** showing 50% displacement ellipsoids. Symmetry code: (i) − *x*, 1 − *y*, 1 − *z*.

The complete molecule of compound **19** (Figure 14) is generated by crystallographic inversion symmetry in space group *P*2_1_/*c*. The conformation of the C1—N3—C5—C6 fragment of the linking chain is *gauche* [82.68 (11)°] as is the N3—C5—C6—C6^i^ (i = −*x*, 1 − *y*, 1 − *z*) fragment [64.96 (13)°], but C5—C6—C6^i^—C5^i^ is of course constrained to be *anti* [180.0°] by symmetry. The N3–H1n grouping lies to the same side of the molecule as N1.

**Figure 15:**
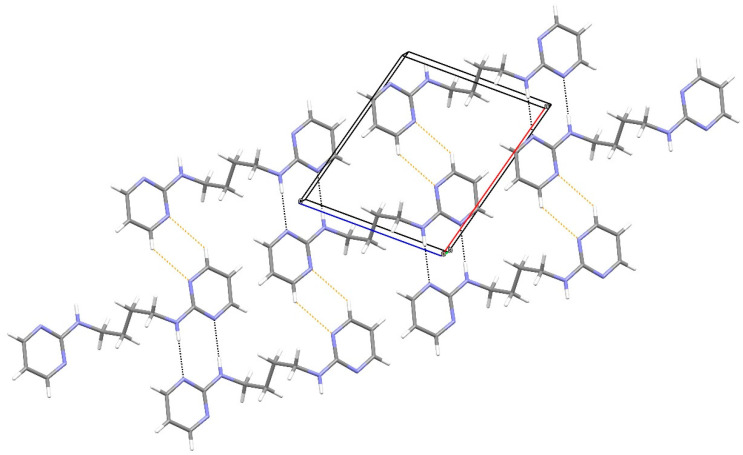
Fragment of a (010) supramolecular sheet in compound **19** showing the primary N–H…N hydrogen bonds (black dashed lines) and secondary C–H…N links (orange dashed lines). The red and blue components of the unit-cell outline indicate the crystallographic [100] and [001] directions, respectively.

In the crystal of compound **19**, hydrogen-bonded chains (or supramolecular tapes) propagating in the [001] direction are formed by pairwise N3–H1n^…^N1 hydrogen bonds involving the NH group and the adjacent N1 atom of the pyrimidine ring, which generate centrosymmetric $R_{2}^{2}$(8) loops [39]. When pairwise weak C4–H4^…^N2 interactions are considered [also $R_{2}^{2}$(8) loops], then (010) supramolecular sheets (Figure 15) arise.

**Figure 16:**
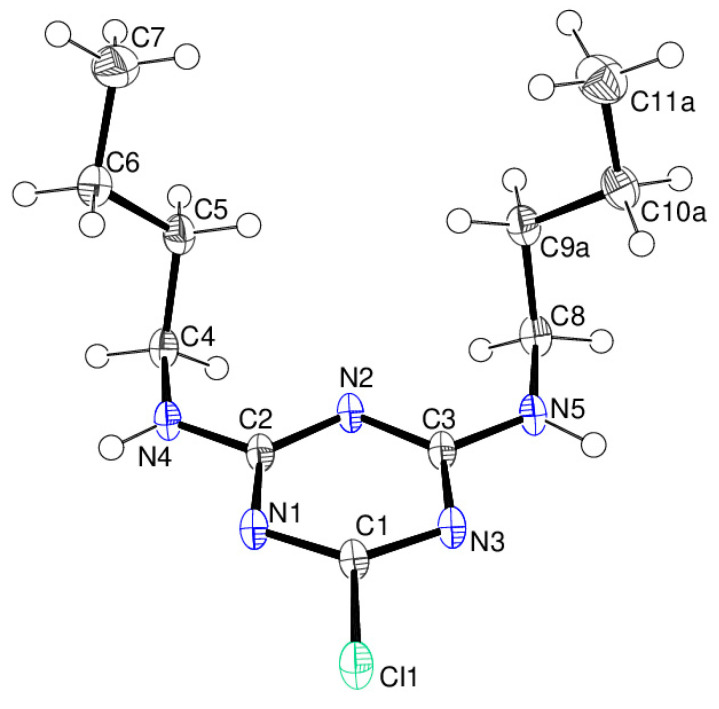
The molecular structure of compound **12** showing 50% displacement ellipsoids. Only one disorder component of the C8–C11 butyl chain is shown.

The molecular structure of compound **12** is shown in Figure 16. There is one molecule in the asymmetric unit in the triclinic space group *P* $\bar{1}$. Both pendant *N*-butyl chains project to the same side of the triazine ring. The N4–C7 chain shows a *gauche*–*anti* (*ga*) conformation as indicated by the N4—C4—C5—C6 and C4—C5—C6—C7 torsion angles of −61.76 (17) and 179.76 (15)°, respectively. The N5–C11 chain is disordered over two orientations in a 0.579 (5):0.421 (5) ratio, but both disorder components also show a *ga* conformation [N5—C8—C9a—C10a = 68.3 (2)°; C8—C9a—C10a—C11a = −168.0 (3)°; N5—C8—C9b—C10b = −68.6 (3)°; C8—C9b—C10b—C11b = 174.3 (4)°]. Compound **12** is a polymorph of the structure reported by Aldilla et al. [25] with reference code KEFLUK in the Cambridge Structural Database [40], which was recrystallised from trichloromethane solution. In KEFLUK both butyl arms have *anti*–*anti* conformations and are disordered. It is notable that compound **12** [*V* = 667.35 (5) Å^3^; ρ = 1.283 g cm^−3^] is significantly denser than KEFLUK [*V* = 731.93 (14) Å^3^; ρ = 1.170 g cm^−3^], which suggests that compound **12** (recrystallised from DCM/light petroleum ether) can pack more efficiently and is the more stable polymorph.

**Figure 17:**
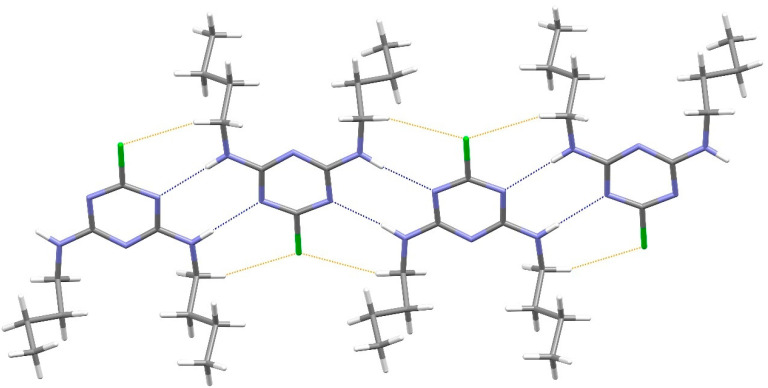
Fragment of a [110] hydrogen-bonded supramolecular tape in the extended structure of compound **12**. The primary N–H^…^N and secondary C–H^…^Cl hydrogen bonds are shown as blue and orange dashed lines, respectively.

In the extended structure of compound **12**, supramolecular tapes propagating in the [110] direction occur (Figure 17). ‘Self-complementary’ [25] N–H…N hydrogen bonds link adjacent molecules with the same $R_{2}^{2}$(8)-loop *motif* as seen in compound **19**. Here, the chain is reinforced by secondary C–H…Cl hydrogen bonds [H^…^Cl = 2.92 and 2.94 Å]. The chain motif in KEFLUK [40] is essentially the same as that in **12** with the same space group and similar unit-cell parameters.

**Figure 18:**
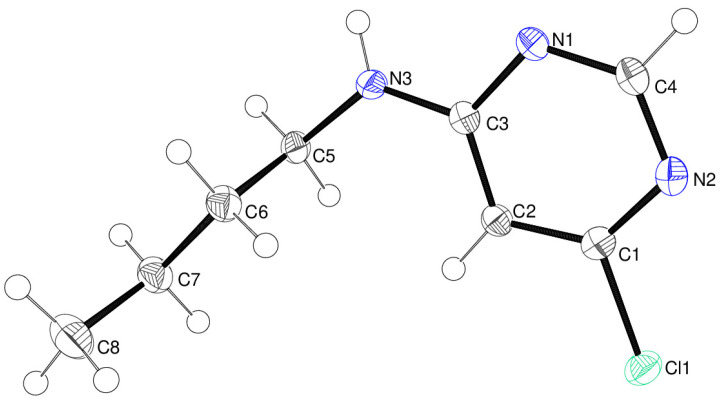
The molecular structure of compound **20** showing 50% displacement ellipsoids.

**Figure 19:**
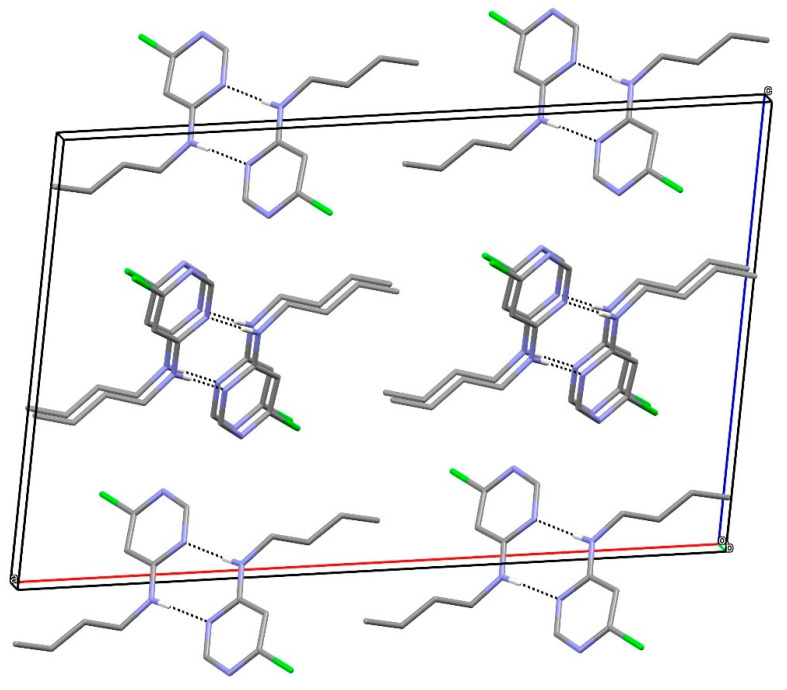
The unit-cell packing for compound **20** viewed down [010]. Carbon-bound hydrogen atoms are omitted for clarity and hydrogen bonds are shown as black dashed lines. The red and blue components of the unit-cell outline indicate the crystallographic [100] and [001] directions, respectively.

Compound **20** (space group *C*2/*c*) crystallises with one molecule in the asymmetric unit (Figure 18). The *N*-butyl chain is extended [N3—C5—C6—C7 = −174.12 (10)°; C5—C6—C7—C8 = −178.14 (11)°]. In the extended structure of compound **20** (Figure 19), inversion dimers are formed via pairwise N3–H1n^…^N1^ii^ (ii = ½−*x*, ½−*y*, 1–*z*) hydrogen bonds with an $R_{2}^{2}$(8)-loop *motif*, but there is no further connectivity into a chain as seen for compound **12**. The packing of the dimers is illustrated in Figure 19.

**Figure 20:**
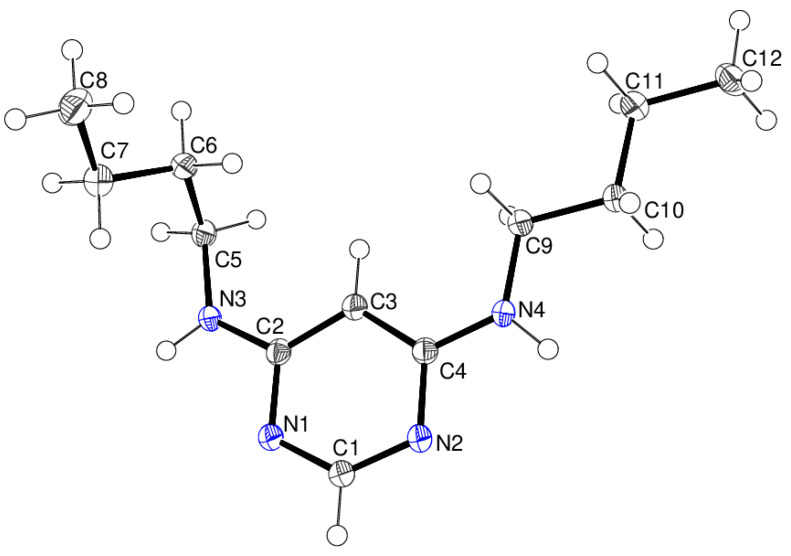
The molecular structure of compound **14** showing 50% displacement ellipsoids.

In compound **14**, which crystallises with one asymmetric molecule (Figure 20) in space group *P*2_1_/*c*, the *N*-butyl arms (including their ring carbon atoms) have different conformations: the C2–C8 chain is *gauche–gauche–anti*, as evidenced by torsion angles of −79.39 (15), −58.58 (14) and 179.90 (11)° for C2—N3—C5—C6, N3—C5—C6—C7 and C5—C6—C7—C8, respectively. Conversely, the C4–C12 chain is *anti–anti–anti* [C4—N4—C9—C10 = 179.32 (11)°; N4—C9—C10—C11 = 177.66 (11)°; C9—C10—C11—C12 = −178.38 (11)°].

**Figure 21:**
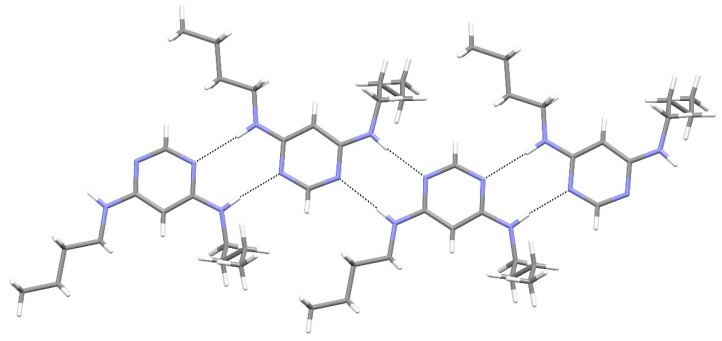
Fragment of a [010] hydrogen-bonded supramolecular tape in the extended structure of compound **14** with N–H^…^N hydrogen bonds shown as black dashed lines.

The extended structure of compound **14** (Figure 21) features the same $R_{2}^{2}$(8)-loop hydrogen-bonded supramolecular tapes that were seen in compound **16**, although here, the tapes propagate in the [010] direction and there are no identified reinforcing weak contacts.

**Figure 22:**
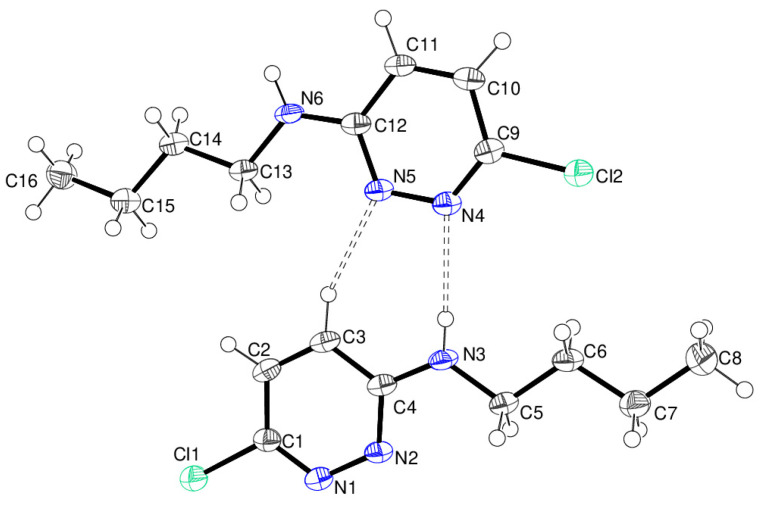
The molecular structure of compound **21** showing 50% displacement ellipsoids. Hydrogen bonds are shown as double-dashed lines.

**Figure 23:**
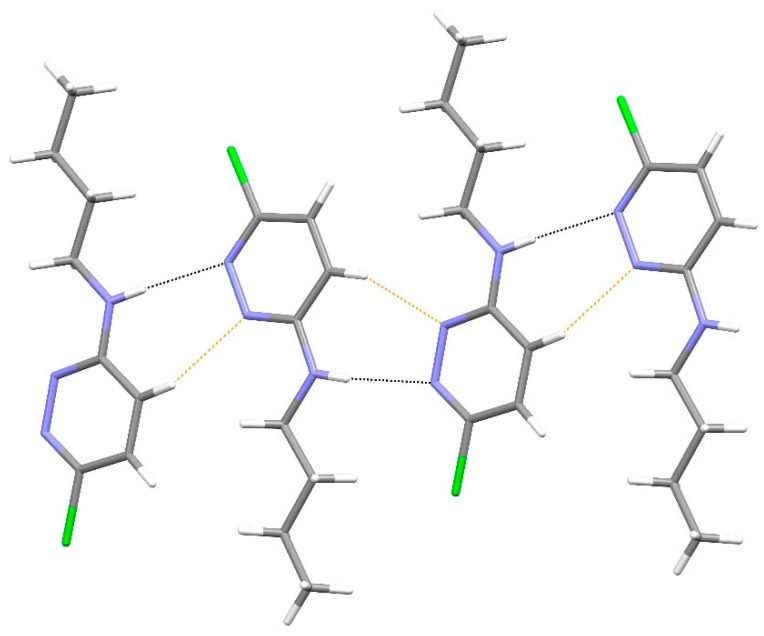
Fragment of a [110] hydrogen-bonded supramolecular tape in the extended structure of compound **21** with N–H^…^N and C–H^…^N hydrogen bonds shown as black and orange dotted lines, respectively.

Compound **21** (Figure 22) crystallises with two molecules in the asymmetric unit in space group *P*$\bar{1}$. The conformations of the C1 and C9 molecules are very similar and the *N*-butyl chains are extended [minimum torsion angle modulus = 171.91 (19)°]. In the crystal, [110] chains of alternating C1 and C9 molecules arise (Figure 23) with each molecule donating an N–H^…^N and adjacent C–H^…^N hydrogen bond to its neighbour.

**Figure 24:**
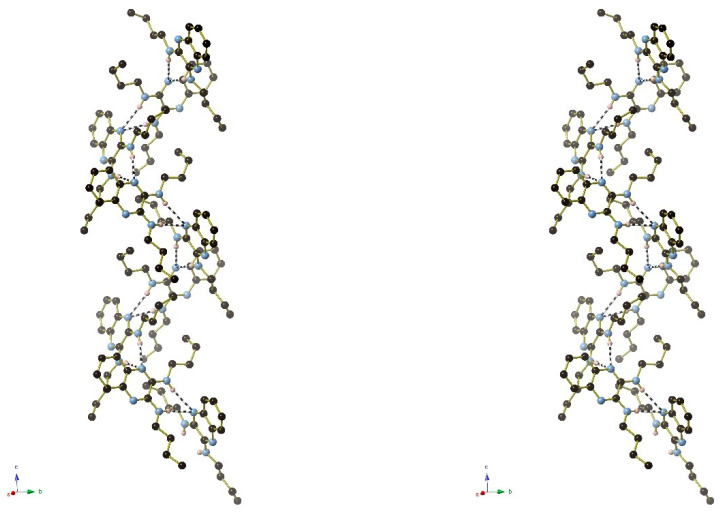
Stereoview of compound **16**.

**Figure 25:**
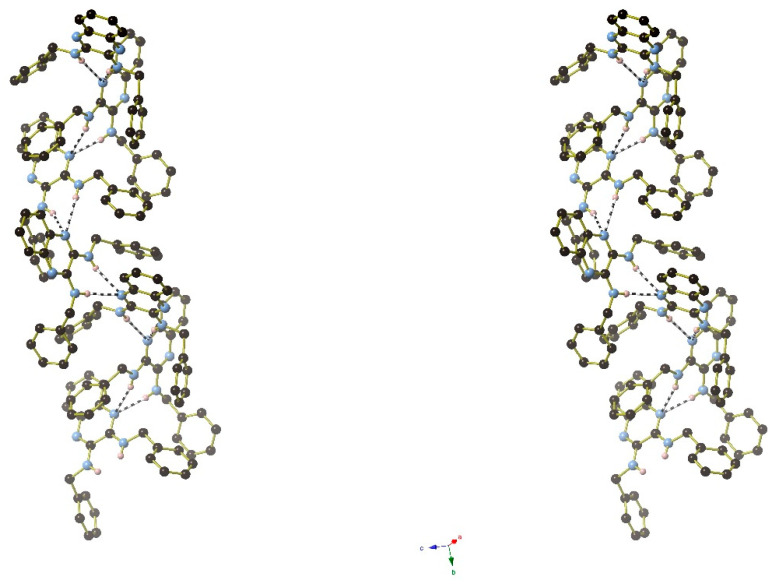
Stereoview of compound **17**.

**Figure 26:**
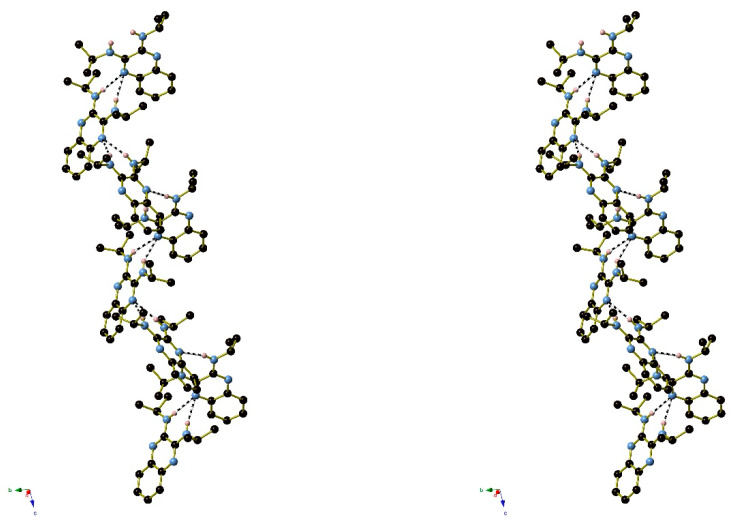
Stereoview of compound **18**.

Figure 24, Figure 25 and Figure 26 are stereoviews together for compounds **16**–**18**. These allow a clearer view of the helical axis for each one and the chain symmetry can be seen and compared (4_1_, 2_1_ and 3_1_). To view the images in 3D, use a pokescope or stereoviewer on a print. Alternatively, view the images on a mobile phone. Look up close blurring of the images but creating one image, then slowly move the phone or page away to bring the 3D image into focus.

## 3. Crystal Structure Determinations

The crystal structures described above were established using intensity data collected on a Rigaku CCD diffractometer. The structures were routinely solved by dual-space methods using SHELXT [41] and the structural models were completed and optimized by refinement against |*F*|^2^ with SHELXL-2018 [42]. The N-bound hydrogen atoms were located in difference maps and their positions were freely refined; the C-bound H atoms were placed in idealized locations (C–H = 0.95–0.99 Å depending on hybridisation) and refined as riding atoms. Methyl groups, if present, were allowed to rotate, but not to tip, to best fit the electron density. The constraint *U*_iso_(H) = 1.2*U*_eq_(carrier) or 1.5U_eq_(methyl carrier) was applied in all cases. The molecular graphics were rendered with *ORTEP*3 [43] and *Mercury* [44]. Full details of the structures and refinements are available in the deposited cifs.

Crystal data for **16** C_16_H_24_N_4_ (pale brown block 0.36 × 0.19 × 0.17 mm), *M*_r_ = 272.39, tetragonal, space group *I*4_1_/*a* (No. 88), *a* = 20.3010 (3) Å, *c* = 14.7609 (4) Å, *V* = 6083.4 (2) Å^3^, *Z* = 16, *T* = 100 K, Mo Kα radiation, λ = 0.71073 Å, μ = 0.073 mm^−1^, ρ_calc_ = 1.190 g cm^−3^, 41,804 reflections measured (3.4 ≤ 2θ ≤ 55.0°), 3491 unique (*R*_Int_ = 0.016), *R*(*F*) = 0.045 [3222 reflections with *I* > 2σ(*I*)], *wR*(*F*^2^) = 0.123 (all data), Δρ_min,max_ (*e* Å^−3^) = −0.35, +0.39, CCDC deposition number 2392272.

Crystal data for **17** C_22_H_20_N_4_ (colourless rod 0.26 × 0.05 × 0.04 mm), *M*_r_ = 340.42, monoclinic, space group *P*2_1_/*c* (No. 14), *a* = 9.8317 (3) Å, *b* = 16.8795 (4) Å, *c* = 22.0694 (8) Å, β = 99.560 (3)°, *V* = 3611.64 (19) Å^3^, *Z* = 8, *Z*′ = 2, *T* = 100 K, Cu Kα radiation, λ = 1.54184 Å, μ = 0.594 mm^−1^, ρ_calc_ = 1.252 g cm^−3^, 38,240 reflections measured (6.6 ≤ 2θ ≤ 132.0°), 6287 unique (*R*_Int_ = 0.048), *R*(*F*) = 0.040 [5327 reflections with *I* > 2σ(*I*)], *wR*(*F*^2^) = 0.099 (all data), Δρ_min,max_ (*e* Å^−3^) = −0.19, +0.20, CCDC deposition number 2392273.

Crystal data for **18** C_14_H_20_N_4_ (colourless rod 0.25 × 0.05 × 0.03 mm), *M*_r_ = 244.34, trigonal, space group *P*3_1_ (No. 144), *a* = 9.5576 (2) Å, *c* = 25.6490 (6) Å, *V* = 2029.08 (10) Å^3^, *Z* = 6, *Z*′ = 2, *T* = 100 K, Cu Kα radiation, λ = 1.54184 Å, μ = 0.581 mm^−1^, ρ_calc_ = 1.200 g cm^−3^, 20,069 reflections measured (10.3 ≤ 2θ ≤ 139.8°), 4220 unique (*R*_Int_ = 0.034), *R*(*F*) = 0.029 [4040 reflections with *I* > 2σ(*I*)], *wR*(*F*^2^) = 0.075 (all data), Δρ_min,max_ (*e* Å^−3^) = −0.17, +0.17, Flack absolute structure parameter 0.10 (16), CCDC deposition number 2392274.

Crystal data for **19** C_12_H_16_N_6_ (colourless plate 0.18 × 0.13 × 0.02 mm), *M*_r_ = 244.31, monoclinic, space group *P*2_1_/*c* (No. 14), *a* = 9.4233 (4) Å, *b* = 8.5179 (3) Å, *c* = 7.9616 (3) Å, β = 104.728 (4)°, *V* = 618.05 (4) Å^3^, *Z* = 2, *Z*′ = ½, *T* = 100 K, Cu Kα radiation, λ = 1.54184 Å, μ = 0.688 mm^−1^, ρ_calc_ = 1.313 g cm^−3^, 6064 reflections measured (9.7 ≤ 2θ ≤ 140.9°), 1175 unique (*R*_Int_ = 0.018), *R*(*F*) = 0.028 [1071 reflections with *I* > 2σ(*I*)], *wR*(*F*^2^) = 0.072 (all data), Δρ_min,max_ (*e* Å^−3^) = −0.16, +0.24, CCDC deposition number 2392275.

Crystal data for **12** C_11_H_20_ClN_5_ (yellow plate 0.26 × 0.10 × 0.02 mm), *M*_r_ = 257.77, triclinic, space group *P*$\bar{1}$ (No. 2), *a* = 5.28500 (19) Å, *b* = 9.8409 (4) Å, *c* = 13.4153 (6) Å, α = 78.554 (3)°, β = 82.696 (3)°, γ = 78.512 (3)°, *V* = 667.35 (5) Å^3^, *Z* = 2, *T* = 100 K, Mo Kα radiation, λ = 0.71073 Å, μ = 0.274 mm^−1^, ρ_calc_ = 1.283 g cm^−3^, 17,919 reflections measured (4.3 ≤ 2θ ≤ 55.0°), 3073 unique (*R*_Int_ = 0.047), *R*(*F*) = 0.054 [2727 reflections with *I* > 2σ(*I*)], *wR*(*F*^2^) = 0.156 (all data), Δρ_min,max_ (*e* Å^−3^) = −0.41, +0.83, CCDC deposition number 2392276.

Crystal data for **20** C_8_H_12_ClN_3_ (colourless block 0.17 × 0.08 × 0.03 mm), *M*_r_ = 185.66, monoclinic, space group *C*2/*c* (No. 15), *a* = 24.4401 (4) Å, *b* = 4.99796 (9) Å, *c* = 15.5982 (3) Å, β = 98.822 (2)°, *V* = 1882.79 (6) Å^3^, *Z* = 8, *T* = 100 K, Cu Kα radiation, λ = 1.54184 Å, μ = 3.181 mm^−1^, ρ_calc_ = 1.310 g cm^−3^, 17,610 reflections measured (7.3 ≤ 2θ ≤ 147.3°), 1882 unique (*R*_Int_ = 0.025), *R*(*F*) = 0.025 [1767 reflections with *I* > 2σ(*I*)], *wR*(*F*^2^) = 0.066 (all data), Δρ_min,max_ (*e* Å^−3^) = −0.21, +0.23, CCDC deposition number 2392277.

Crystal data for **14** C_12_H_22_N_4_ (colourless rod 0.32 × 0.05 × 0.02 mm), *M*_r_ = 222.33, monoclinic, space group *P*2_1_/*c* (No. 14), *a* = 5.3655 (2) Å, *b* = 12.1195 (4) Å, *c* = 19.3616 (6) Å, β = 92.309 (3)°, *V* = 1258.01 (7) Å^3^, *Z* = 4, *T* = 100 K, Cu Kα radiation, λ = 1.54184 Å, μ = 0.568 mm^−1^, ρ_calc_ = 1.174 g cm^−3^, 12,050 reflections measured (8.7 ≤ 2θ ≤ 142.1°), 2398 unique (*R*_Int_ = 0.048), *R*(*F*) = 0.038 [1914 reflections with *I* > 2σ(*I*)], *wR*(*F*^2^) = 0.099 (all data), Δρ_min,max_ (*e* Å^−3^) = −0.25, +0.15, CCDC deposition number 2392278.

Crystal data for **21** C_8_H_12_ClN_3_ (colourless plate 0.12 × 0.10 × 0.02 mm), *M*_r_ = 185.66, triclinic, space group *P*$\bar{1}$ (No. 2), *a* = 5.29107 (13) Å, *b* = 7.52230 (19) Å, *c* = 24.2953 (7) Å, α = 89.285 (2)°, β = 85.299 (2)°, γ = 73.370 (2)°, *V* = 923.35 (4) Å^3^, *Z* = 4, *Z*′ = 2, *T* = 100 K, Cu Kα radiation, λ = 1.54184 Å, μ = 3.243 mm^−1^, ρ_calc_ = 1.336 g cm^−3^, 16,660 reflections measured (3.7 ≤ 2θ ≤ 140.3°), 3452 unique (*R*_Int_ = 0.033), *R*(*F*) = 0.039 [2933 reflections with *I* > 2σ(*I*)], *wR*(*F*^2^) = 0.104 (all data), Δρ_min,max_ (*e* Å^−3^) = −0.46, +0.34, CCDC deposition number 2392279.

## 4. Materials and Methods

IR spectra were recorded on an Everest diamond-attenuated total reflection (ATR) Fourier transform infrared (FTIR) spectrometer (Thermo Fischer Scientific, 1 Ashley Road, Altrincham, UK); ultraviolet (UV) spectra were recorded using an Evolution Perkin-Elmer Lambda 25 UV-Vis spectrometer with EtOH as the solvent (Perkin-Elmer LAS Chalfont Road, Seer Green, Beaconsfield, HP9 2FX, UK). The term sh means shoulder; ^1^H and ^13^C nuclear magnetic resonance (NMR) spectra were recorded at 400 and 100.5 MHz, respectively, using a Bruker 400 spectrometer (Bruker UK Ltd., Welland House, Westwood Business Park, Coventry, CV4 8HZ, UK). Chemical shifts, δ, are given in ppm and measured by comparison with the residual solvent. Coupling constants, *J*, are given in Hz; high-resolution mass spectra were obtained at the University of Wales, Swansea, using an Atmospheric Solids Analysis Probe (ASAP) (positive mode) instrument: Xevo G2-S ASAP (Waters Ltd., Stamford Avenue, Altrincham Road, Wilmslow, SK9 4AX, UK); melting points were determined on a Cole-Palmer MP-200D Stuart digital melting point apparatus (9 Orion Court, Ambuscade Road, Colmworth Business Park, St Neots, Cambridgeshire, PE19 8YX, UK).

The synthesis of compound **16** was reported in a previous paper [1].

Compound **17**

2,3-Dichloroquinoxaline (300 mg, 1.5 mmol) in EtOH (10 mL) was mixed with benzylamine (323 mg, 3.0 mmol) and Et_3_N (304 mg, 3.0 mmol) and sealed in a flat-lid PTFE Parr pressure vessel. The vessel was heated at 150 °C in a circular aluminium block for 18 h on a hotplate in a fume hood. After cooling, the contents were diluted with water (200 mL) in a beaker and extracted with DCM (100 mL × 2). The combined extracts were dried with MgSO_4_, filtered and evaporated to dryness. The product was purified by chromatography on silica. Elution with DCM followed by DCM/Et_2_O [80:20] gave the *title compound* (174 mg, 34%) as colourless crystals, m.p. 180–181 °C (from dichloromethane–light petroleum ether). ν_max_ (cm^−1^) 3304w, 1590w, 1549s, 1494s, 1452s, 1302s, 1201s, 750s, 695s, 638s, 603s, 588s and 466s; δ_H_ (400 MHz; CDCl_3_) 4.75 (4H, s), 4.88–5.06 (2H, s, NH), 7.25–7.42 (12H, m) and 7.73 (2H, dd, *J* = 8.0 and 4.0); δ_C_ (100.1 MHz; CDCl_3_) 45.9, 125.1, 125.6, 127.7, 128.6, 128.7, 136.8, 138.5 and 143.9; *m*/*z* (Orbitrap ASAP) 341.1763 (M^+^ + H, 100%) C_22_H_20_N_4_H requires 341.1766

Compound **18**

2,3-Dichloroquinoxaline (300 mg, 1.5 mmol) in EtOH (10 mL) was mixed with isopropylamine (178 mg, 3.0 mmol) and Et_3_N (304 mg, 3.0 mmol) and sealed in a flat-lid PTFE Parr pressure vessel. The vessel was heated at 150 °C in a circular aluminium block for 18 h on a hotplate in a fume hood. After cooling, the contents were diluted with water (200 mL) in a beaker and extracted with DCM (100 mL × 2). The combined extracts were dried with MgSO_4_, filtered and evaporated to dryness. The product was purified by chromatography on silica. Elution with DCM followed by DCM/Et_2_O [90:10] gave the *title compound* (169 mg, 46%) as colourless crystals, m.p. 200–201 °C (from dichloromethane–light petroleum ether). ν_max_ (cm^−1^) 3343w, 2971w, 1596w, 1556s, 1489s, 1456s, 1364s, 1296s, 1210s, 1164s, 1125s, 935w, 747s, 602s, 499s and 439s; δ_H_ (400 MHz; CDCl_3_) 1.35 (12H, d, *J* = 8.0), 4.26–4.36 (2H, NH), 4.41–4.50 (2H, m), 7.32 (2H, dd, *J* = 8.0 and 4.0) and 7.66 (2H, dd, *J* = 8.0 and 4.0); δ_C_ (100.1 MHz; CDCl_3_) 22.7, 42.8, 124.5, 125.5, 136.7 and 143.6; *m*/*z* (Orbitrap ASAP) 245.1766 (M^+^ + H, 100%) C_14_H_20_N_4_H requires 245.1766.

Compound **19**

2-Chloropyrimidine (300 mg, 2.62 mmol) in acetonitrile (30 mL) was treated with 1,4-diaminobutane (115 mg, 1.31 mmol) and Et_3_N (265 mg, 2.62 mmol). The mixture was heated for 18 h at 85 °C. After cooling, it was diluted with water (200 mL) and extracted twice with DCM (100 mL). The combined extracts were dried over MgSO_4_ and evaporated. The mixture was purified by chromatography on silica. The column was eluted with DCM followed by Et_2_O then Et_2_O/MeOH (90:10) to give the *title compound* (35 mg, 11%) as clear crystals, m.p. 189–190 °C (from dichloromethane–light petroleum ether). ν_max_ (cm^−1^) 3247w, 2937w, 1575s, 1539s, 1459s, 1412s, 1367s, 1345s, 1278s, 1228s, 1182s, 1112s, 1088s, 800s, 730s, 642s, 614s, 538s, 509s and 406s; δ_H_ (400 MHz; D_6_DMSO) 1.50–1.57 (4H, m), 3.22–3.28 (4H, m), 6.52 (2H, t, *J* = 5.0), 7.09–7.16 (2H, m, NH), 8.21 (4H, d, *J* = 5.0); δ_C_ (100.1 MHz; D_6_DMSO) 26.9, 40.9, 110.1, 158.3 and 162.7; *m*/*z* (Orbitrap ASAP) 245.1510 (M^+^ + H, 100%) C_12_H_16_N_6_H requires 245.1515

Compounds **12** [25] and **22** [31].

Cyanuric chloride (200 mg, 1.09 mmol) in CH_3_CN (30 mL) was treated with BuNH_2_ (238 mg, 3.26 mmol) and Et_3_N (329 mg, 3.26 mmol) with stirring. The mixture was heated at 85 °C for 20 h. After cooling, the mixture was diluted with water (200 mL) and extracted twice with DCM (100 mL). The combined extracts were dried over MgSO_4_, filtered and evaporated. TLC showed that two polar products were present eluting with DCM/Et_2_O (80:20). Elution with DCM/Et_2_O (80:20) gave the *title compound* **12** (140 mg, 54%) as transparent plates, m.p. > 200 °C (from dichloromethane–light petroleum ether). ν_max_ (cm^−1^) 3251w, 3098w, 2957w, 2931w, 2872, 2858w, 1633s, 1544vs, 1403vs, 1359vs, 1311s, 1279s, 1115s, 1097s, 985s, 796vs, 733s, 564w, 447w and 410w; δ_H_ (400 MHz; CDCl_3_) 0.88 (12H, t, J = 6.0), 1.24–1.34 (8H, m), 1.41–1.52 (8H, m), 3.19 (4H, q, *J* = 6.0) and 3.23 (4H, q, J = 6.0); δ_C_ (100.1 MHz; CDCl_3_) 14.0, 14.1, 14.2, 19.9, 31.3, 165.4, 165.8, 166.0, 167.9 and 168.5 (11 peaks are overlapping); *m*/*z* (Orbitrap ASAP) 258.1480 (M^+^ + H, 100%) C_11_H_20_N_5_ClH requires 258.1486. Elution with DCM/Et_2_O (60:40) gave the *title compound* **22** (30 mg, 9%) as an oil: ν_max_ (cm^−1^) 3269w, 2955w, 2928w, 2870w, 1560vs, 1501vs, 1422vs, 1351vs, 1263w, 1224w, 1172w, 1128w, 811vs, 734w and 501w; δ_H_ (400 MHz; CDCl_3_) 0.84 (9H, t, *J* = 7.0), 1.28 (6H, h, *J* = 7.0), 1.43 (6H, q, *J* = 7.0), 3.26 (6H, s) and 4.81–5.12 (3H, s, NH); δ_C_ (100.1 MHz; CDCl_3_) 13.7, 20.1, 32.1, 40.1 and 166.1

Compounds **20** and **14** [27,28]

4,6-Dichloropyrimidine (300 mg, 2.0 mmol) in CH_3_CN (10 mL) was mixed with BuNH_2_ (294 mg, 4.0 mmol) and Et_3_N (407 mg, 4.0 mmol) and heated at 130 °C in a 23 mL Parr pressure vessel for 18h. Upon cooling, the mixture was diluted with water (200 mL), extracted with DCM (100 mL), dried over MgSO_4_, filtered and evaporated. Two products were purified by chromatography on silica. Elution with DCM then Et_2_O gave the *title compound* **20** (114 mg, 31%) as colourless crystals, m.p. 85–86 °C (from dichloromethane–light petroleum ether). ν_max_ (cm^−1^) 3234w, 3095w, 3024w, 2953w, 2926w, 2869w, 1600s, 1570s, 1535s, 1451s, 1393s, 1336s, 1235w, 1199w, 1087s, 983s, 8903, 772s, 744s, 585s, 549w, 457w and 416w; δ_H_ (400 MHz; CDCl_3_) 0.97 (3H, t, *J* = 6.0), 1.42 (2H, s, *J* = 6.0), 1.62 (2H, q, *J* = 6.0), 3.28 (2H, s, br), 5.39–5.78 (1H, s, br), 6.36 (1H, s) and 8.32 (1H, s); δ_C_ (100.1 MHz; CDCl_3_) 13.7, 19.8, 31.0, 41.4, 158.0 and 163.0; *m*/*z* (Orbitrap ASAP) 186.0797 (M^+^ + H, 100%) C_8_H_12_N_3_ClH requires 186.0798. Elution with MeOH:Et_2_O (30:70) gave the *title compound* **14** (86 mg, 20%) as crystals, m.p. 160–161 °C (from dichloromethane–light petroleum ether). ν_max_ (cm^−1^) 3228w, 3088w, 2954w, 2930w, 2859w, 1588s, 1426s, 1359s, 1346s, 1260s, 1244s, 1215s, 1136s, 1119s, 1099s, 981s, 793s, 771s, 723s, 700s, 653s, 616s, 530s and 465s; δ_H_ (400 MHz; CDCl_3_) 0.97 (6H, t, *J* = 6.0), 1.44 (4H, s, *J* = 6.0), 1.62 (4H, q, *J* = 6.0), 3.21 (4H, q, *J* = 6.0), 5.03 (2H, s, NH), 5.23 (1H, s) and 8.05 (1H, s); δ_C_ (100.1 MHz; CDCl_3_) 13.9, 20.3, 31.0, 41.4, 157.1 and 162.7; *m*/*z* (Orbitrap ASAP) 223.1919 (M^+^ + H, 100%) C_12_H_22_N_4_H requires 223.1923

Compound **21**

3,6-Dichloropyridazine (200 mg, 1.34 mmol) in EtOH (10 mL) was mixed with BuNH_2_ (196 mg, 2.68 mmol) and Et_3_N (271 mg, 2.68 mmol) in a PTFE lined Parr pressure vessel. The mixture was heated at 150 °C for 18 h. After cooling, the mixture was diluted with water (200 mL), extracted with DCM (100 mL), dried with MgSO_4_, filtered and evaporated. The mixture was purified by chromatography: DCM followed by DCM/Et_2_O (70:30) eluted the *title compound* (98 mg, 39%) as colourless crystals, m.p. 113–114 °C (from dichloromethane–light petroleum ether). ν_max_ (cm^−1^) 3288s, 2955s, 2928s, 2864s, 1608s, 1567s, 1490s, 1447s, 1373w, 1165s, 1129s, 1070s, 907s, 835s, 737s, 697s, 628w, 560s, 532s and 405s; δ_H_ (400 MHz; CDCl_3_) 0.96 (3H, t, *J* = 7.0), 1.44 (2H, h, *J* = 7.0), 1.65 (2H, q, *J* = 7.0), 3.36–3.42 (2H, m), 5.18 (1H, NH), 6.73 (1H, d, *J* = 8.0) and 7.17 (1H, d, *J* = 8.0); δ_C_ (100.1 MHz; CDCl_3_) 13.7, 20.1, 31.3, 42.1, 116.2, 129.1, 146.1 and 158.3; *m/z* (Orbitrap ASAP) 186.0796 (M^+^ + H, 100%) C_8_H_12_N_3_ClH requires 186.0798.

## 5. Conclusions

In summary the X-ray single crystal structures of eight compounds were analysed: **16**–**19**, **12**, **20**, **14** and **21**. All the compounds are densely packed with no porosity and are white (as microcrystalline powders) or colourless crystals. This contrasts with our previous studies on 2,4-alkyl/arylamino nitrobenzene derivatives, which can crystallise with large channels and are yellow owing to nitro group conjugation [45]. The molecular structures of the compounds described here do not possess any unexpected features, although there is some variety in the conformations of the butyl side chains. As far as their extended structures are concerned, compounds **16**, **17** and **18** based on a quinoxaline nucleus, with different *N*-*ortho*-substituents, share essentially the same hydrogen bond ‘pincer’ motif [graph-set motif [39] $R_{2}^{2}$(7)], which generates a chain in each case but with crystallographic symmetries based on different screw axes (4_1_, 2_1_ and 3_1_, respectively). This suggests that the steric crowding of the bulky substituents attached to the rigid fused rings promotes a helical connectivity [the dihedral angles between the quinoxaline ring systems of adjacent molecules are 85.077 (12), 78.05 (3) and 86.40 (6)° for **16**, **17** and **18**, respectively]. Three examples of helicity suggest that the approach is general for this fascinating *motif* and further exploitation may be possible with this design idea. The extended structures of **12** and **14** feature a consistent $R_{2}^{2}$(8)-loop molecular tape motif, which has already been described for related materials [25]. This robust synthon can occur with (**12**) or without (**14**) reinforcing secondary interactions. Structure **19** demonstrates how supramolecular sheets can be formed when a hydrogen-bond synthon (the same *motif* as **12** and **14**) occurs at each end of the molecule, and **20** shows that when one of the NH groups is replaced by a chlorine atom, then only $R_{2}^{2}$(8)-loop dimers can result. Finally, structure **21** shows cooperative N–H^…^N and C–H^…^N bonds leading to chains. The NMR charts are in the Supplementary Material.

## Data Availability

Data is contained within the article or Supplementary Material.

## Supplementary Materials

The following supporting information can be downloaded at: https://www.mdpi.com/article/10.3390/ijms252212329/s1.
