# Supplementary material for: Stereoview Images of Hydrogen-Bonded Quinoxalines with a Helical Axis; Pyrimidines and a Pyridazine That Form Extended Tapes
Source: Int J Mol Sci. 2024 Nov 17;25(22):12329. doi: 10.3390/ijms252212329 (PMC11594637; doi:10.3390/ijms252212329)
Supplement: Supplementary file 1 [file ijms-25-12329-s001.zip › ijms-3312440-supplementary.pdf]

Supplementary

NMR Data Compounds 17-19, 12, 22, 20, 14, 21

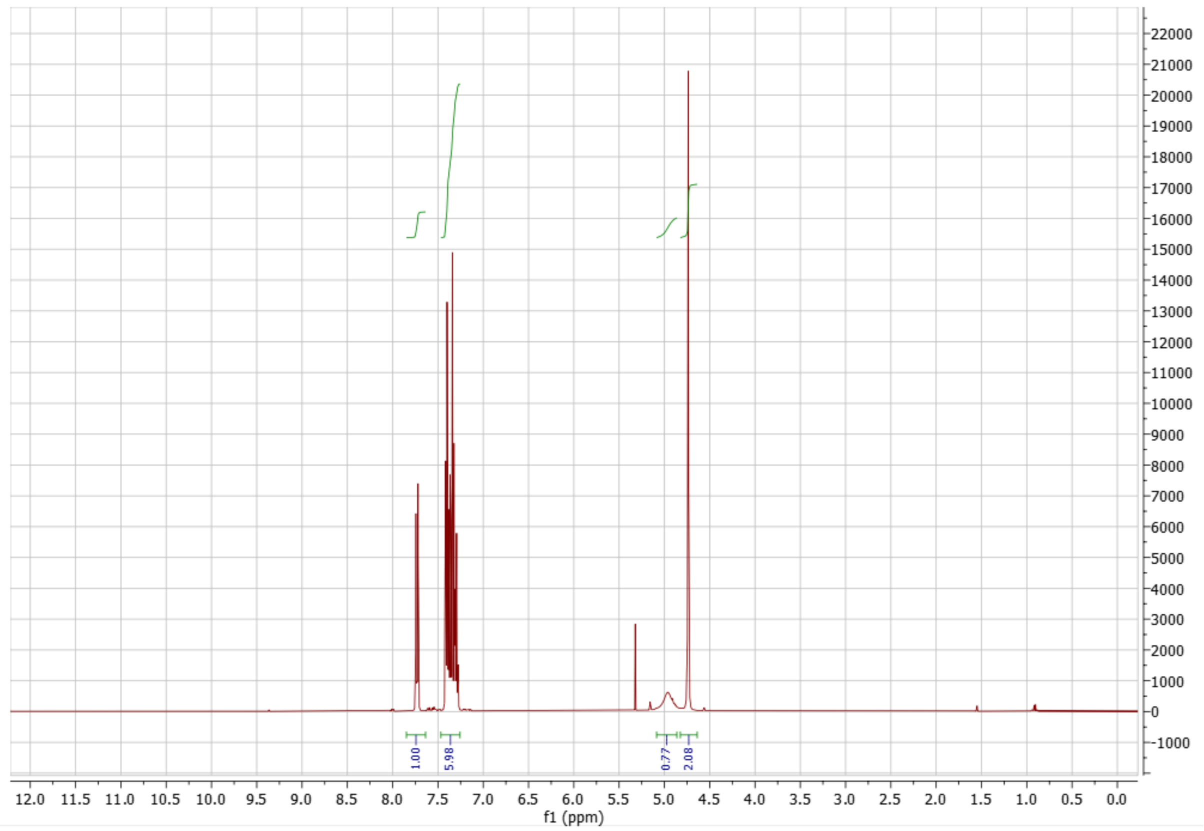

Compound **17** $CDCl_3$ 400MHz Proton NMR $CDCl_3$

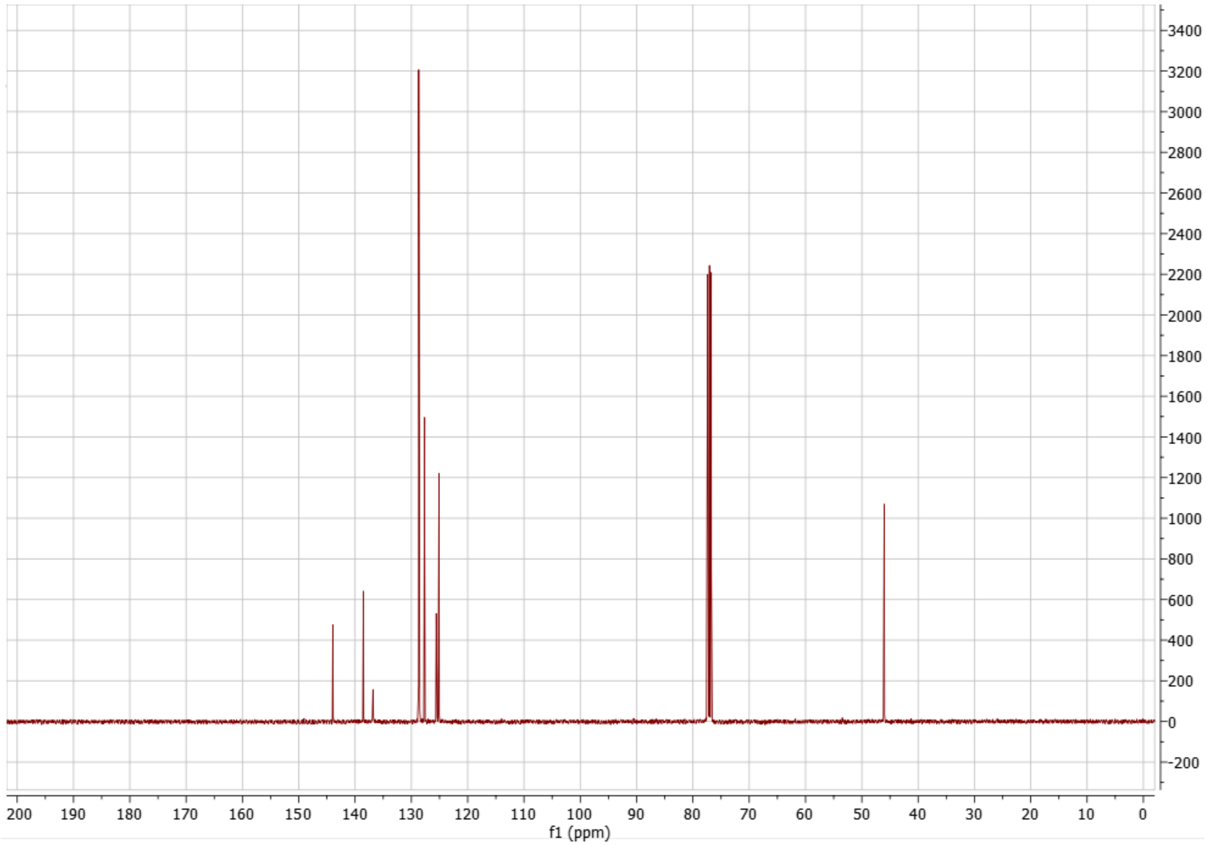

Compound **17** $CDCl_3$ 400MHz Carbon NMR $CDCl_3$

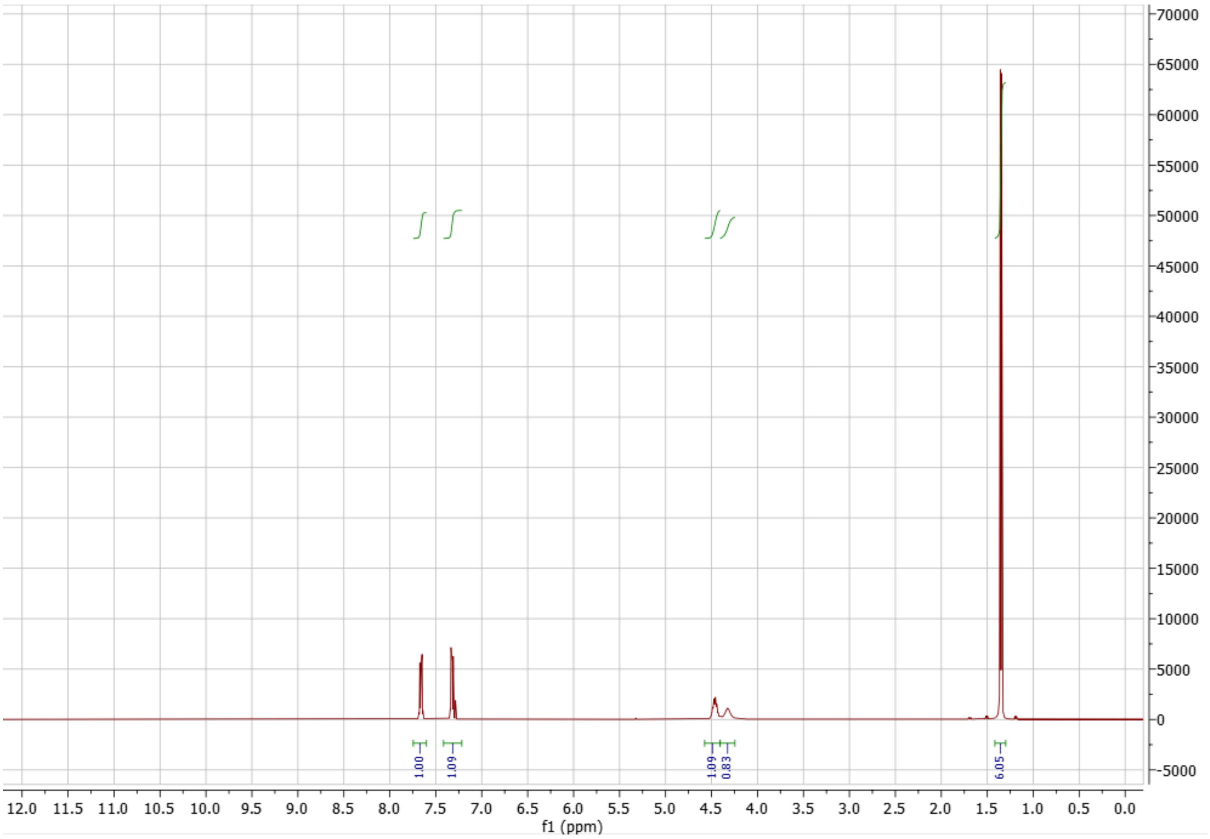

Compound **18** $CDCl_3$ 400MHz Proton NMR $CDCl_3$

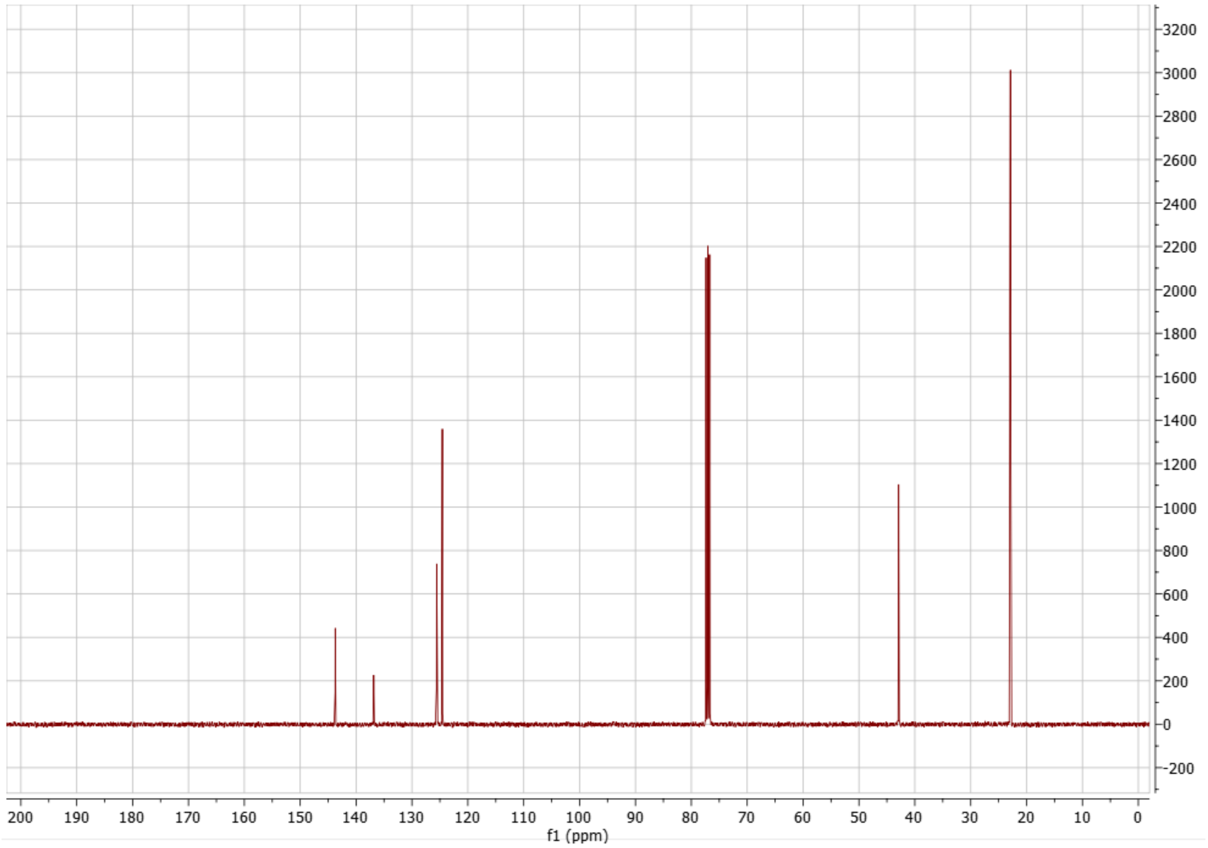

Compound **18** $CDCl_3$ 400MHz Carbon NMR $CDCl_3$

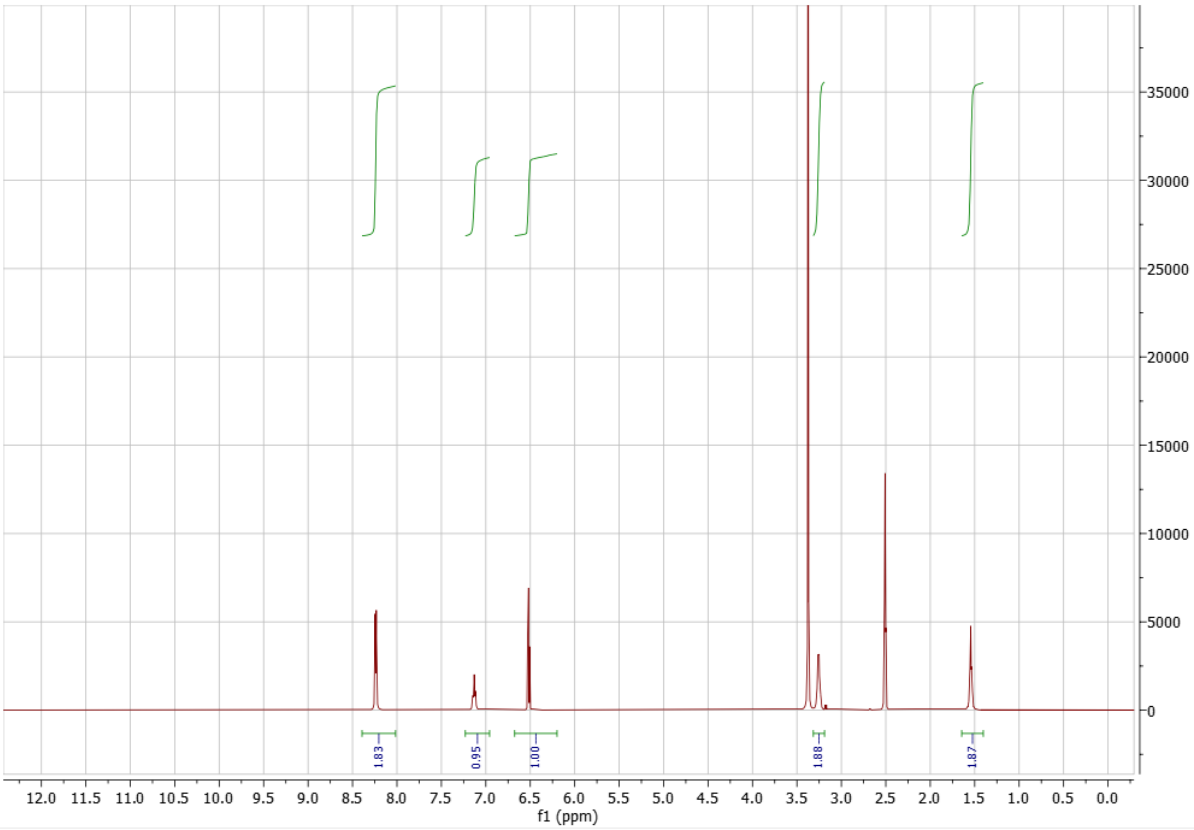

Compound **19** D_6DMSO 400MHz Proton NMR D_6DMSO

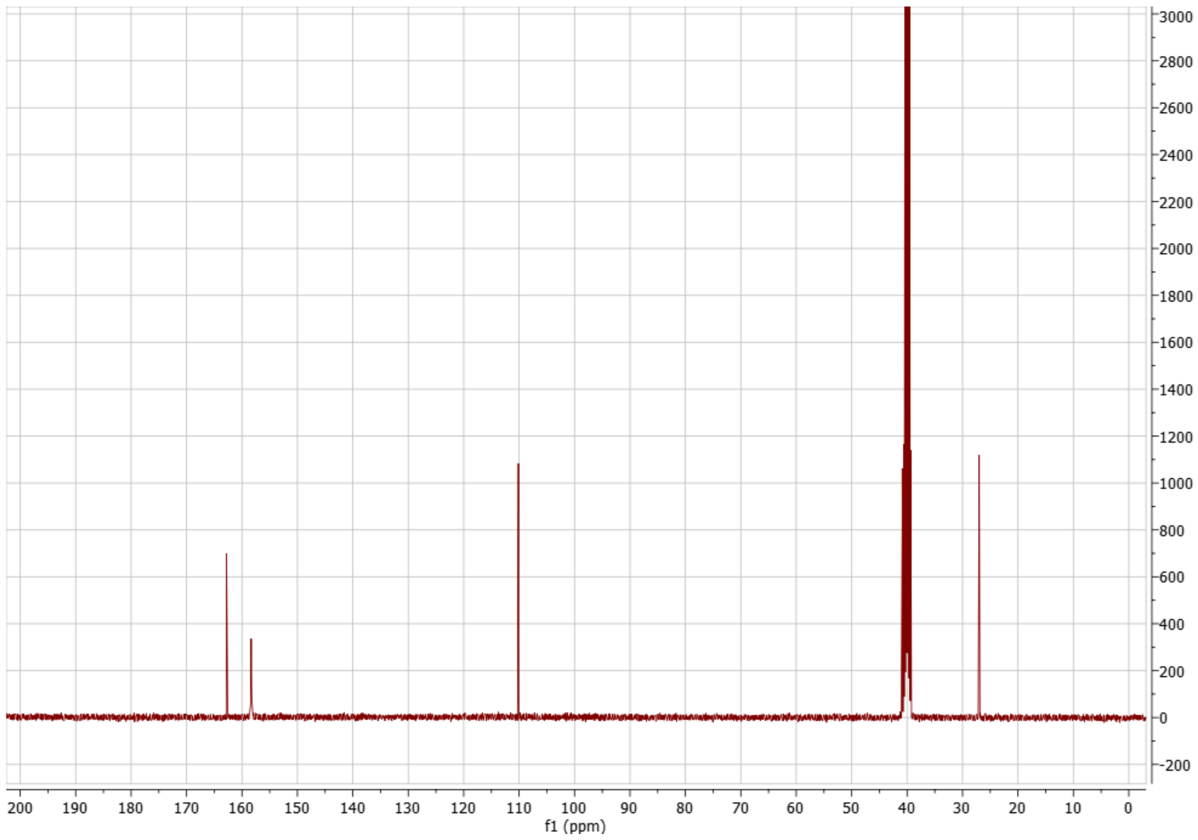

Compound **19** D_6DMSO 400MHz 13 Carbon NMR D_6DMSO

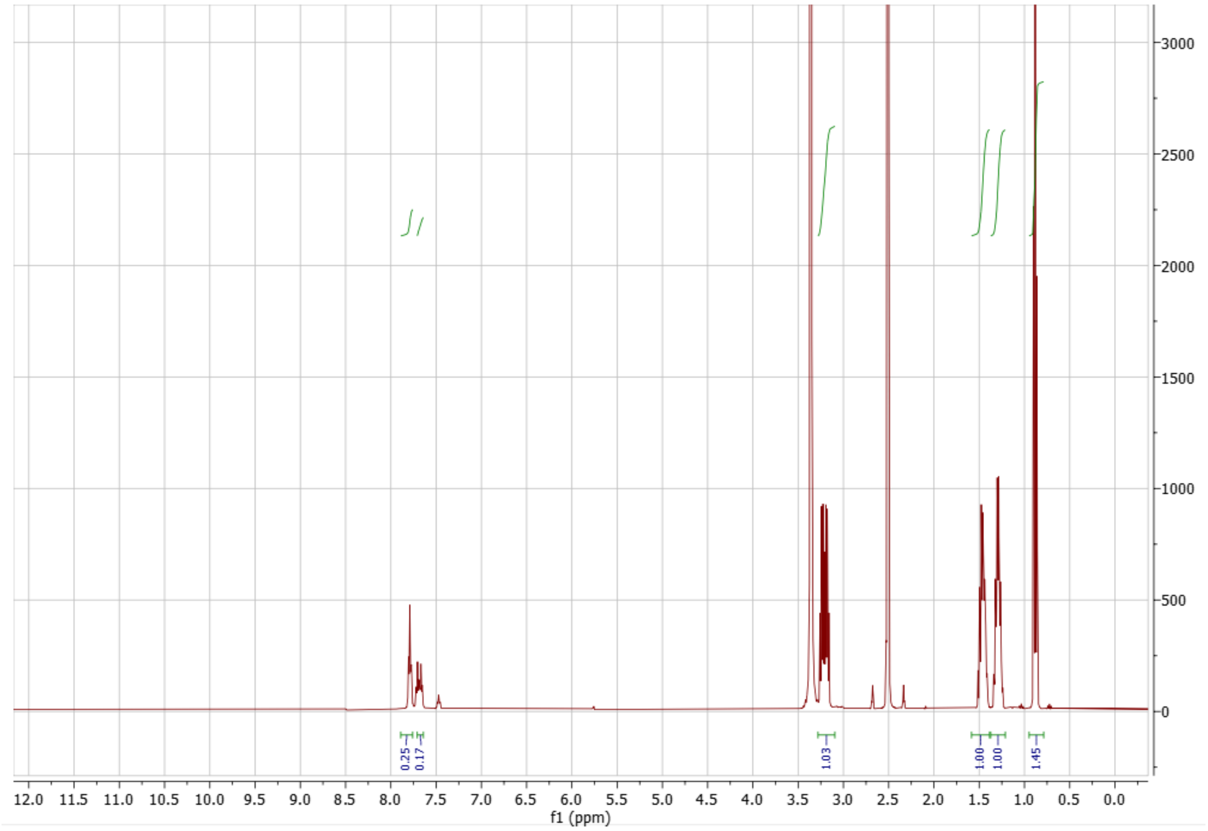

Compound **12** D_6DMSO 400MHz Proton NMR D_6DMSO

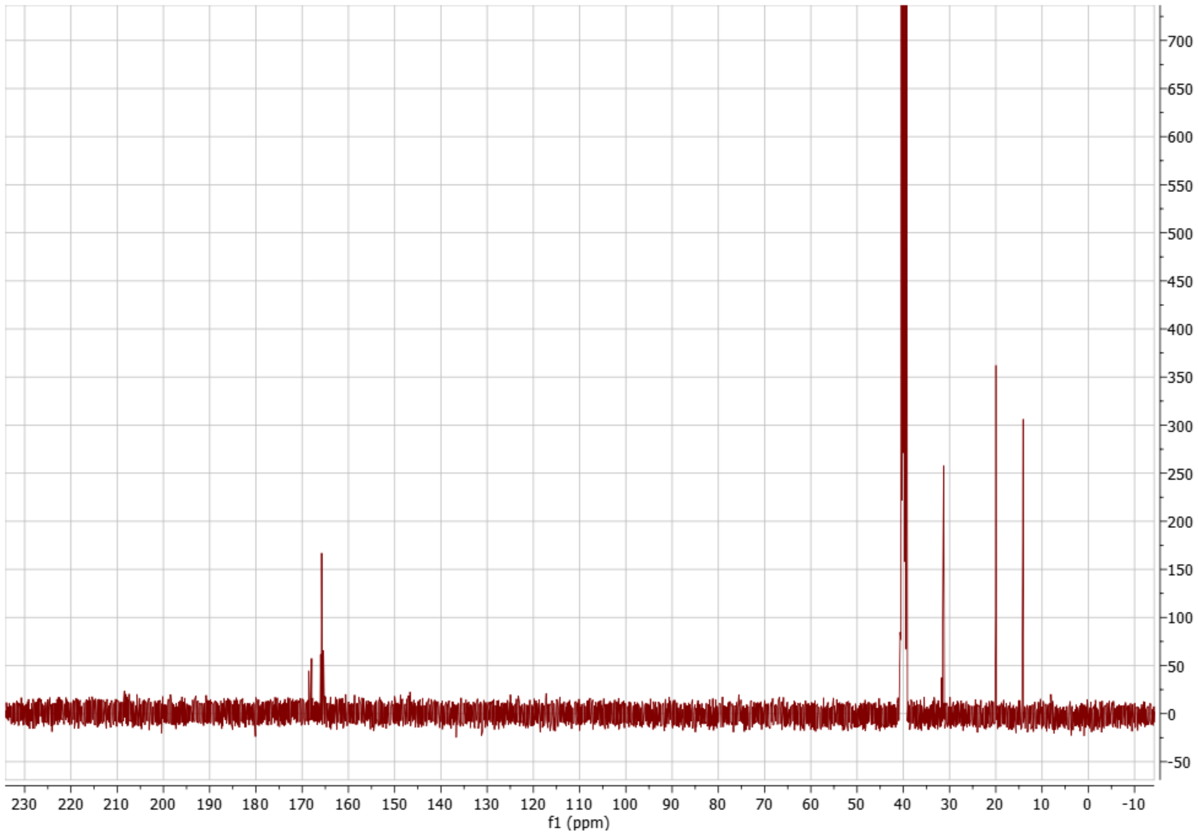

Compound **12** D_6DMSO 400MHz 13 Carbon NMR D_6DMSO

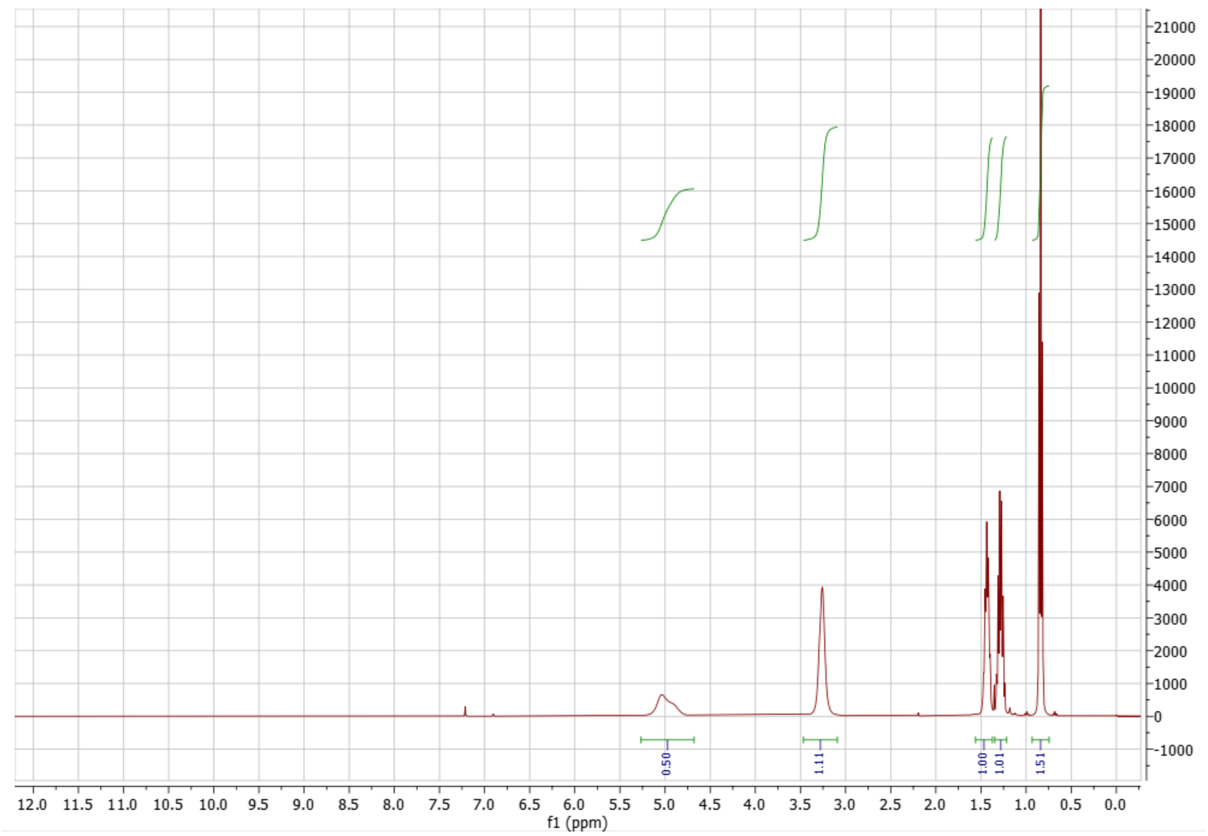

Compound **22** $CDCl_3$ 400MHz Proton NMR $CDCl_3$

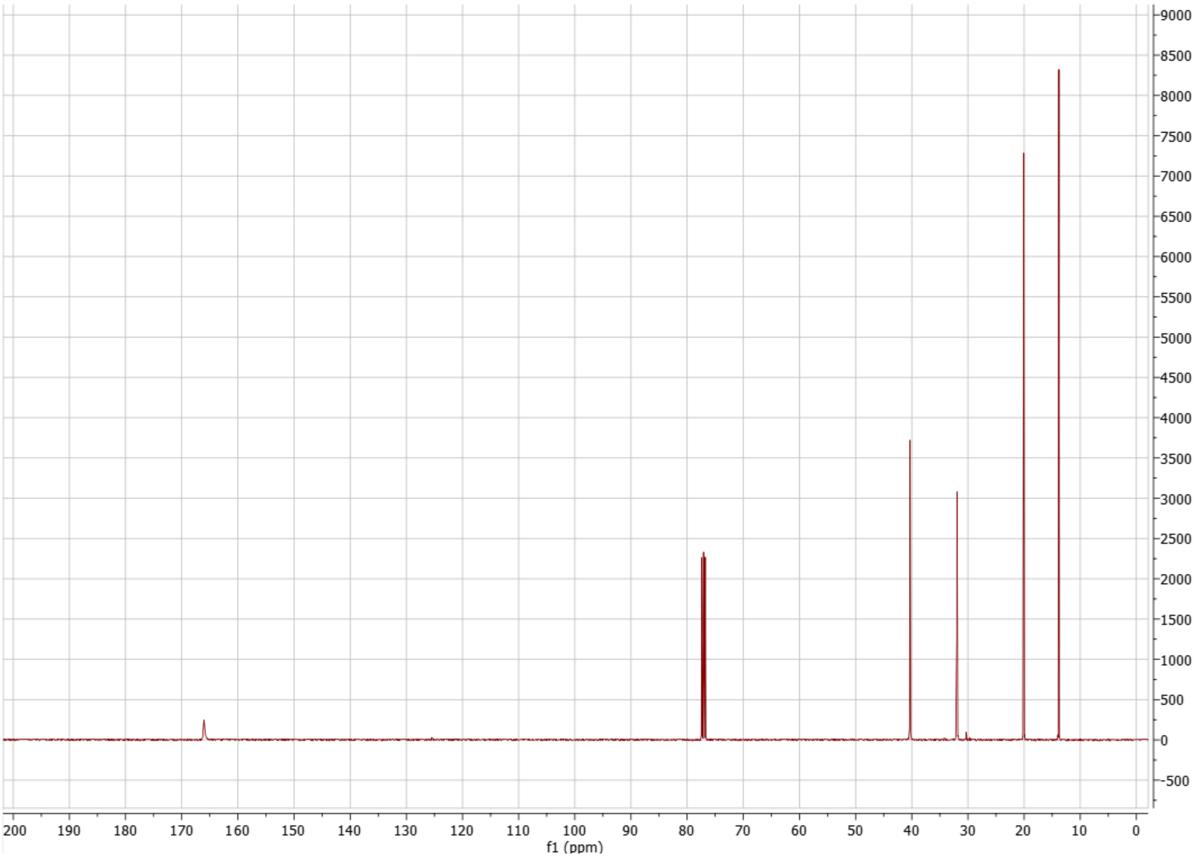

Compound **22** $CDCl_3$ 400MHz 13 Carbon NMR $CDCl_3$

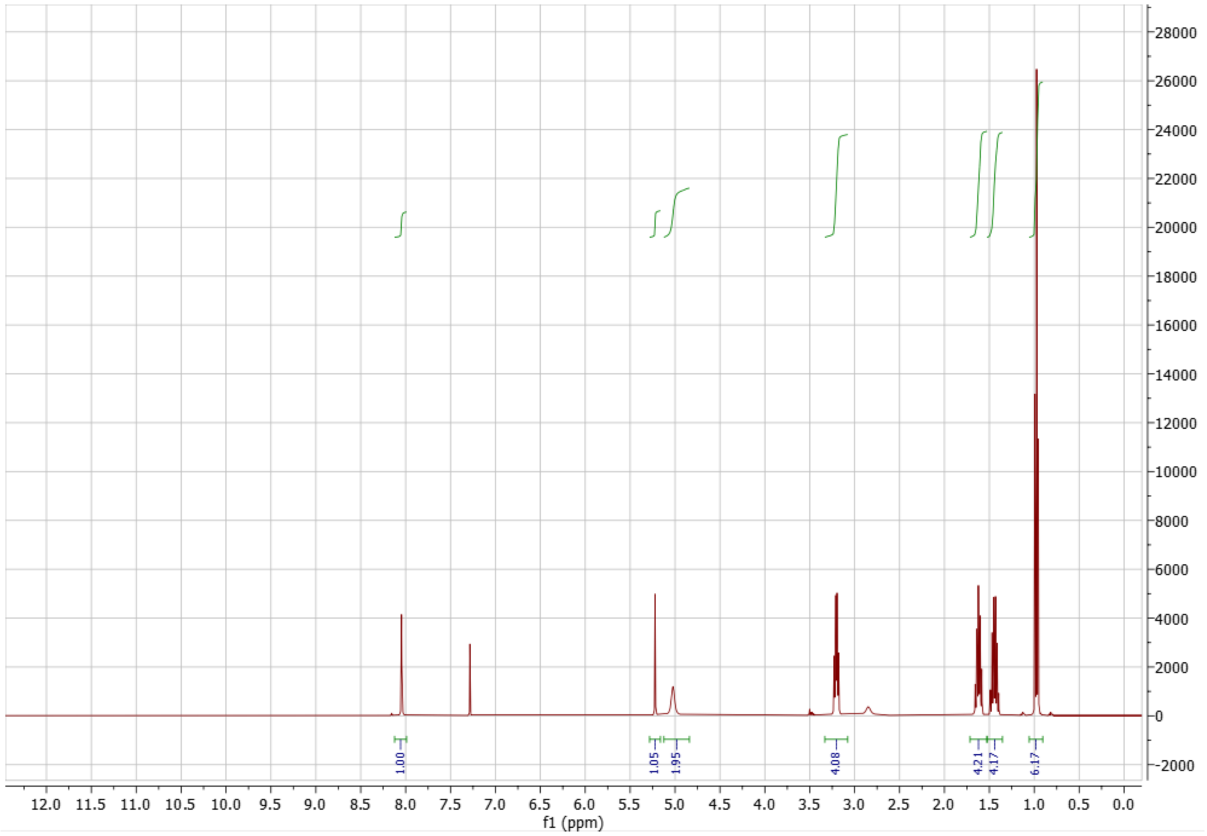

Compound **20** $CDCl_3$ 400MHz Proton NMR $CDCl_3$

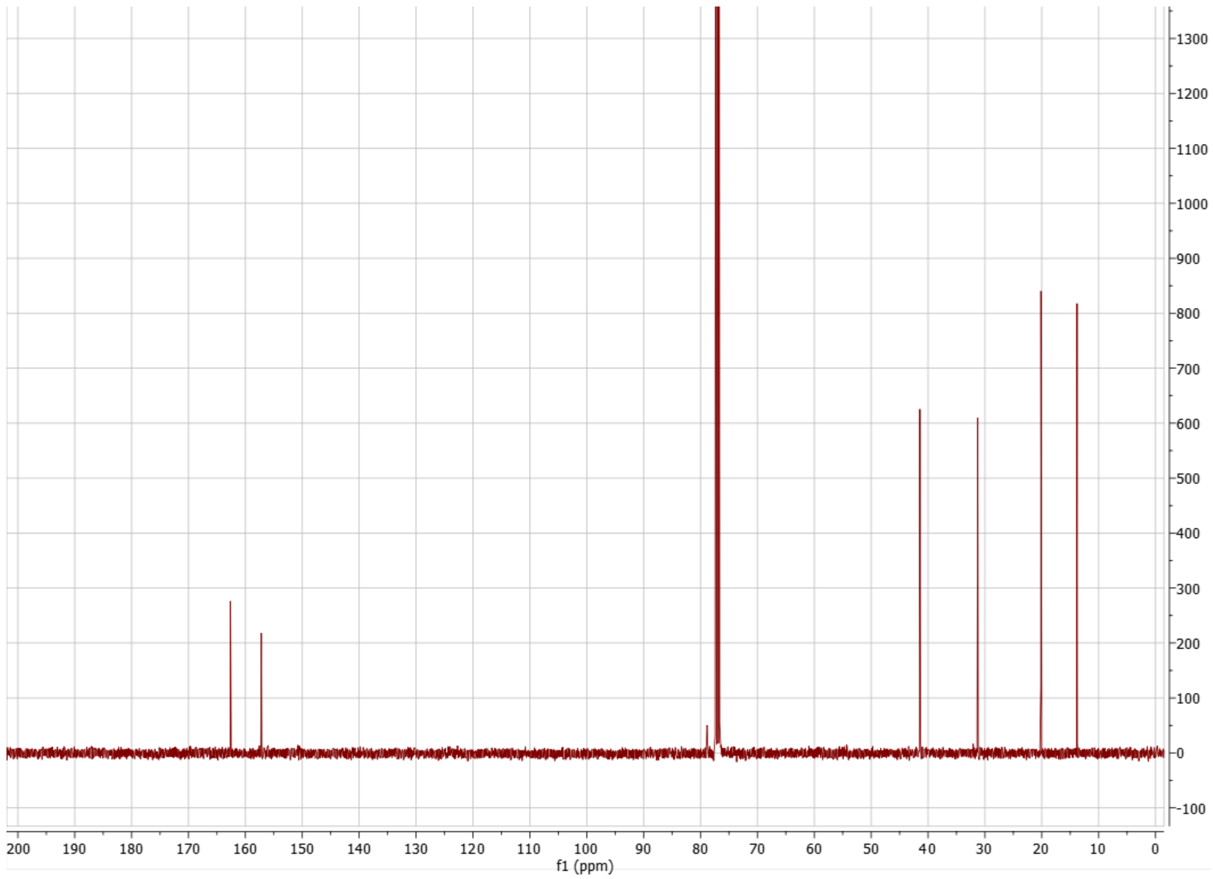

Compound **20** $CDCl_3$ 400MHz 13 Carbon NMR $CDCl_3$

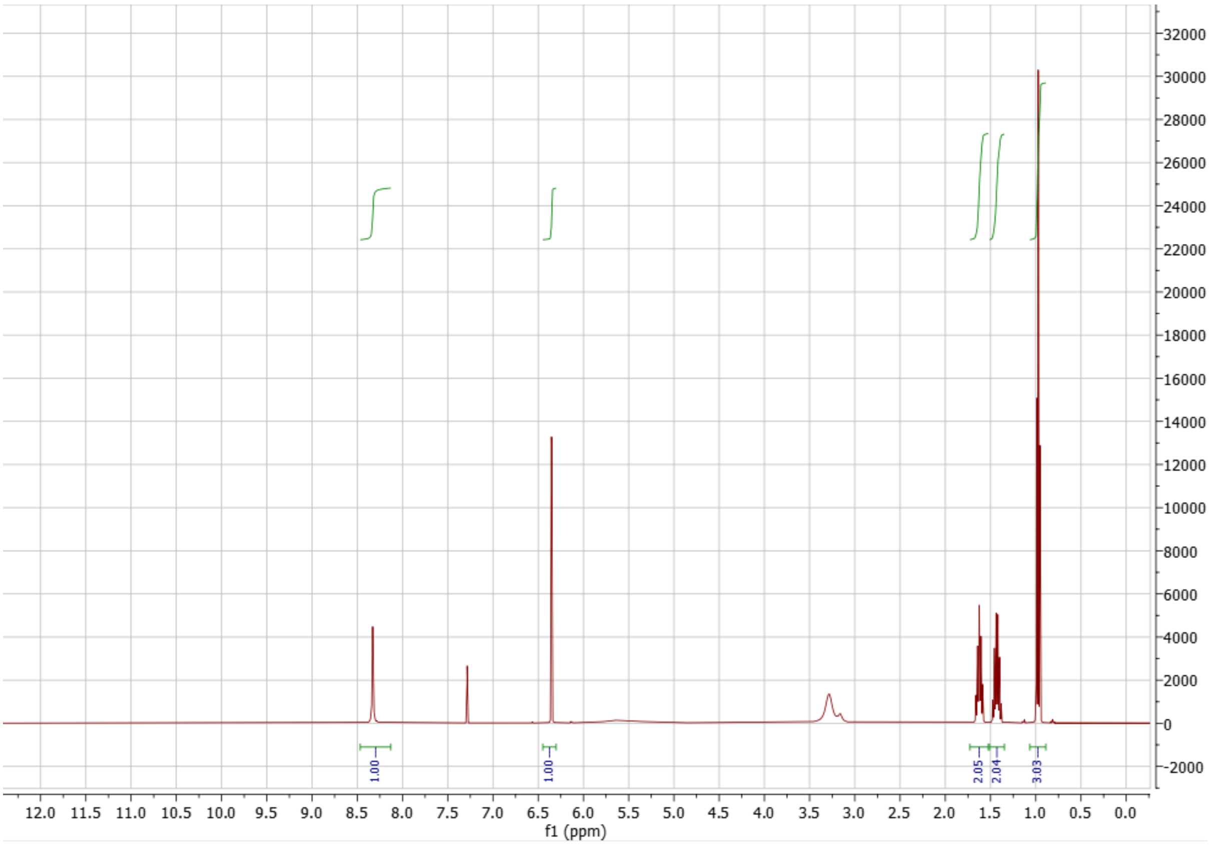

Compound **14** $CDCl_3$ 400MHz Proton NMR $CDCl_3$

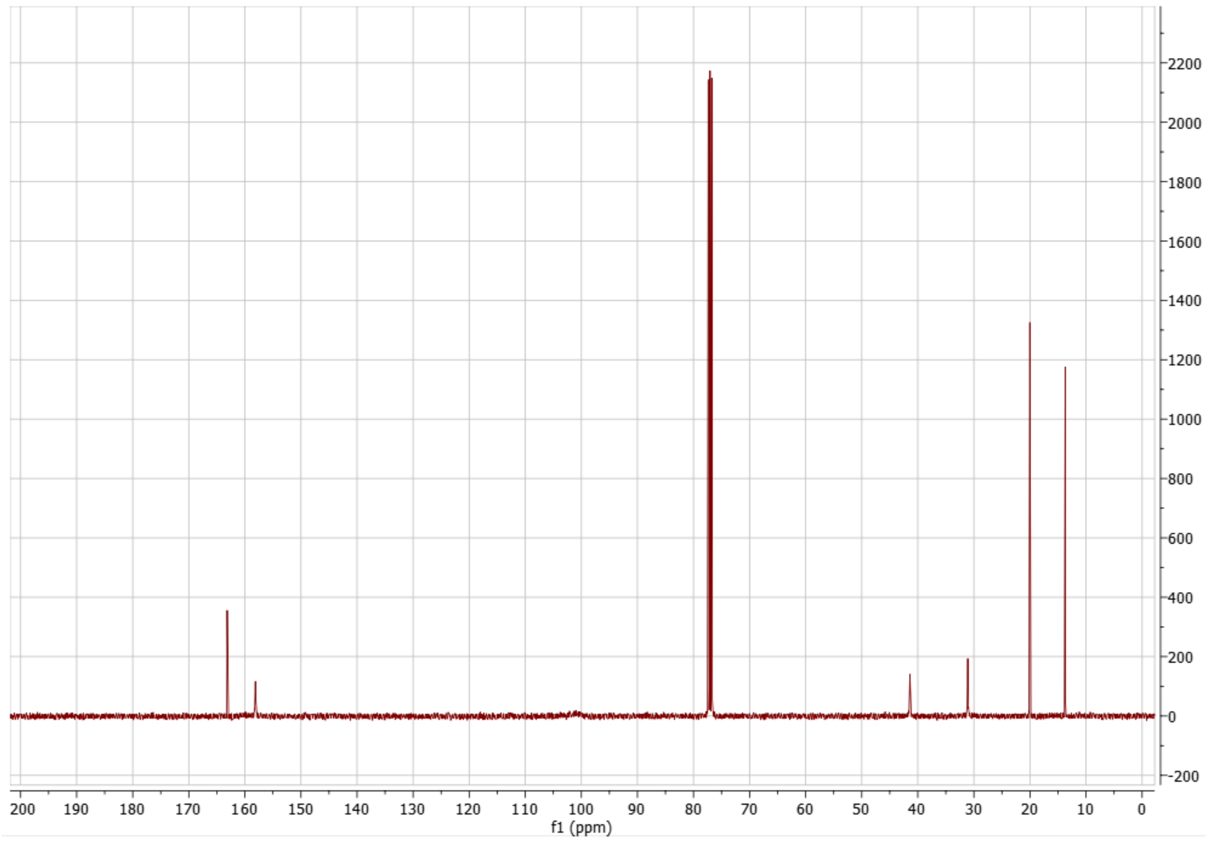

Compound **14** $CDCl_3$ 400MHz 13 Carbon NMR $CDCl_3$

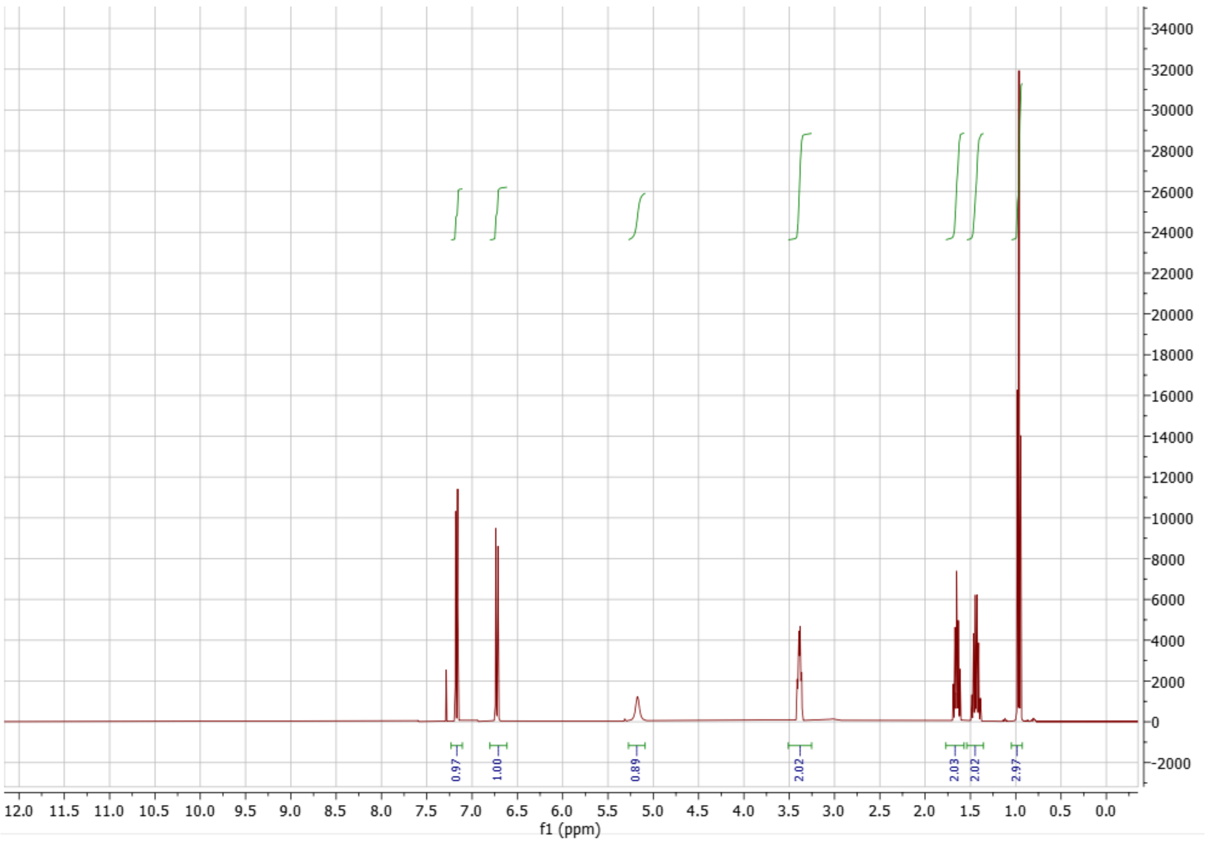

Compound **21** $CDCl_3$ 400MHz Proton NMR $CDCl_3$

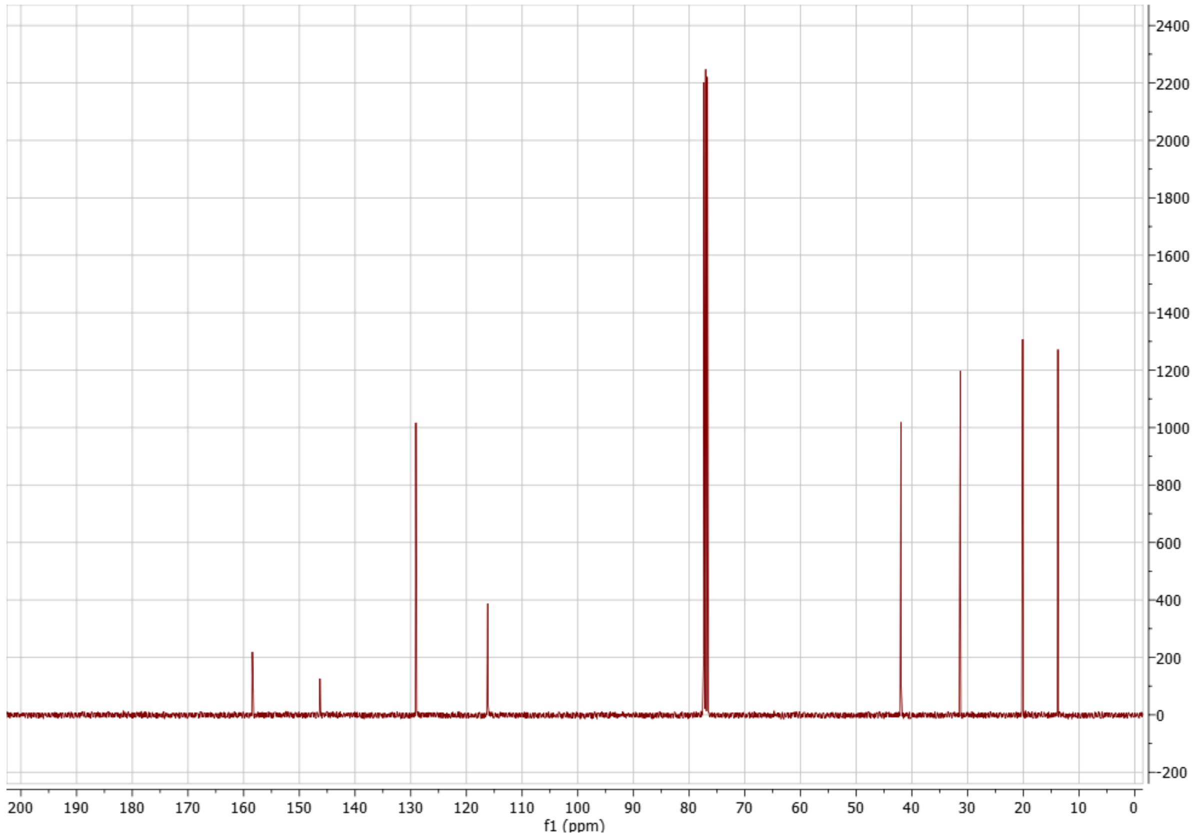

Compound **21** $CDCl_3$ 400MHz 13 Carbon NMR $CDCl_3$
